# The TGF-βR1 inhibitor galunisertib re-shapes the PDAC-TME by limiting decidual-like natural killer cells polarization

**DOI:** 10.1038/s41419-026-08581-9

**Published:** 2026-03-31

**Authors:** Martina Cucchiara, Maria Teresa Palano, Cristian Rubuano, Gianluca De Antoni, Matteo Gallazzi, Daniele Mercatelli, Marta Tagliabue, Adelaide Pessi, Gianpiero Catalano, Maria Gemelli, Riccardo Ricotta, Pier Francesco Ferrucci, Gennaro Nappo, Silvia Uccella, Alessandro Zerbi, Patrizia Borsotti, Giulia Garattini, Federica Di Leva, Nicolò Baranzini, Annalisa Grimaldi, Lorenzo Mortara, Francesco Acquati, Paola Cornelia Muti, Dorina Belotti, Andrea Resovi, Barbara Bassani, Antonino Bruno

**Affiliations:** 1 Laboratory of Innate Immunity, IRCCS MultiMedica, Milan, Italy; 2 Laboratory of Immunology and General Pathology, Department of Biotechnology and Life Sciences, University of Insubria, Varese, Italy; 3 Department of Pharmacy and Biotechnology, University of Bologna, Bologna, Italy; 4 Medical Oncology Department, IRCCS MultiMedica, Milan, Italy; 5 Radiation Oncology Center, IRCCS MultiMedica, Milan, Italy; 6 Pancreatic Surgery Unit, Humanitas Clinical and Research Center-IRCCS, Rozzano, Milan Italy; 7 Department of Biomedical Sciences, Humanitas University, Rozzano, Milan, Italy; 8 Department of Pathology, IRCCS Humanitas Research Hospital, Rozzano, Milan, Italy; 9 Laboratory of Tumor Microenvironment, Department of Oncology, Istituto di Ricerche Farmacologiche Mario Negri IRCCS, Bergamo, Italy; 10 Department of Biotechnology and Life Sciences, University of Insubria, Varese, Italy; 11 Human Genetics Laboratory, Department of Biotechnology and Life Sciences, University of Insubria, Varese, Italy; 12 IRCCS MultiMedica, Milan, Italy; 13 Department of Biomedical, Surgical and Dental Sciences, University of Milan, Milan, Italy

**Keywords:** Pancreatic cancer, Oncogenesis

## Abstract

Pancreatic ductal adenocarcinoma (PDAC) is the third leading cause of cancer-related mortality worldwide. Natural Killer (NKs) cells are pivotal for tumor surveillance but are dysfunctional in PDAC. We evaluated whether pharmacological blockade of TGF-β1/TGF-βR1 axis in PDAC cells and cancer-associated fibroblasts (CAFs) could modulate NK polarization via soluble factors. The phenotype/functions of NKs from PDAC patients versus healthy controls (HC) were compared, and the polarization state of NKs exposed to the conditioned media of PDAC cells and fibroblasts was evaluated by flow cytometry. The ability of galunisertib (GAL) to reverse NK dysfunction in immunocompetent mice orthotopically implanted with FC1199 PDAC cells was evaluated. PDAC patients showed higher TGF-β1/ TGF-βR1 levels than HC, with worse outcomes in TGF-β1^high^/TGF-βR1^high^ patients. Circulating CD9^+^ NKs were expanded in PDAC patients compared with HC and exhibited a pro-angiogenic secretome and higher pro-angiogenic activities in vitro and in vivo (leech Hir*udo verbana*), compared to the CD9^-^ NK cells. PDAC cells and CAF induced a CD9^+^-decidual-like phenotype, also impairing NK degranulation. GAL treatment restrains PDAC cell/CAF-induced NK anergy, restoring their cytotoxicity. Also, TGFβ-R1 knockdown in PDAC cells exhibited the capability to limit the generation of decidual-like NKs, while restoring their antitumor ability, via soluble factors. Secretome profiling of BxPC3 and MIAPaCA2 PDAC cell lines and CAFs showed that GAL downregulated the release of several growth, angiogenic, and immunoregulatory factors, including *FGF2, HGF, IL11, PLGF, EGFR*, and *VEGF*. In vivo in orthotopic tumors formed by FC1199 cells GAL decreased CD9^+^-NK frequency, promoted M1-macrophage polarization, and activated NK and CD8^+^T-cells, together with a significant reduction of tumor weight, fibrosis and inhibition of angiogenesis. Our study identifies CD9^+^NKs as a novel cell subset expanded in PDAC and underscores the role of TGF-β1/TGF-βR1 signalling in promoting a pro-tumoral NKs. GAL-treatment emerges as immunomodulator able in re-educating pro-tumor NKs cells in PDAC.

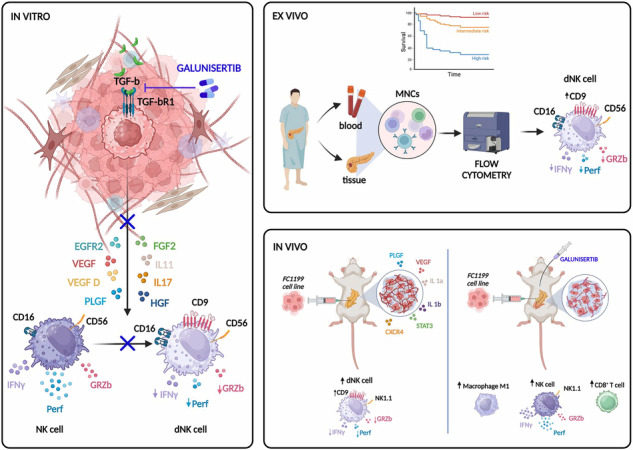

## Introduction

Pancreatic Ductal Adenocarcinoma (PDAC) is the most common malignant neoplasm of the pancreas [1] and is characterized by an immunosuppressive tumor microenvironment (TME) that hampers effective antitumor immune responses [2–4].

Recent studies pointed out on the PDAC subtypes and related heterogeneity, based on molecular analysis, virtual microdissection analysis [5], single-cell and spatial transcriptomic analysis. Based on the molecular alteration, PDAC can be classified into four major classes, namely squamous subtype, progenitor subtype, immunogenic subtype, and aberrantly differentiated endocrine subtype [6]. A further PDAC subtype classification includes the identification of basal-like subtype. Also, considering the relevance of the stromal components of PDAC, it has been possible to define further subtypes, namely normal and activated stromal subtypes [7]. Also, PDAC is characterized by elevated heterogeneity in immune cell composition, spatial distribution and activation states, thus strongly impacting on tumor progression and treatment response [8]. Spatial analysis reveals a heterogenic spatial distribution of TME in PDAC, characterized by the the coexistence of “cold” areas and “hot” neighborhoods areas [8].

Among the different components of the immune tumor microenvironment, PDAC progression has been closely associated with dysfunctional circulating natural killer (NK) cells, characterized by lower expression of activation receptors [9], impaired degranulation capabilities and reduced release of perforin, Granzyme B and IFNγ [10–13].

Recent study also pointed out the interaction, including the spatial analysis, of NK cells with PDAC cellular components [14]. Ouyang et al., by combining single-cell and bulk RNA sequencing, identified an NK cell signature, correlating with immunocyte infiltration, inflammatory reaction, immune checkpoint inhibitors (ICIs) response, in PDAC patients [15]. Go et al. found that PDAC-tissue resident NK cells are able to support patient survival through promotion of cDC1-CD8^+^T cell activity [16].

NKs, representing 5–15% of circulating lymphoid cells in healthy adults, are innate effector lymphocytes, primarily involved in the antiviral and antitumoral response [17, 18]. Two main subsets have been identified according to CD56 and CD16 surface antigen expression [17, 18]. In physiological conditions, circulating NKs are predominantly cytotoxic CD56^dim^CD16^+^, while cancer patients are characterized by the expansion of the CD56^bright^CD16^-^ pro-tumor/anergic subset. A third subset has been recognized within the developing decidua, namely decidual NK cells (dNKs), characterized by the expression of the tetraspanin CD9, and playing a crucial role in the vascularization and tolerance toward embryo [19, 20]. In addition to NK cell reduced antitumor activities in diverse cancers, several studies in the literature [21–23] showed that tumor infiltrating and circulating NKs undergo the angiogenic switch, upregulating the expression of CD9, thereby acquiring the CD56^bright^CD16^-^CD9^+^CD49a^+^NKG2D^low^ decidual-like phenotype in lung [24], colorectal [25], prostate cancer [26], and pleural effusions from metastatic tumors [27].

TGF-β1 has been shown to exert a crucial role [28, 29], downregulating IFNγ production [30] and the expression of activatory receptors [31], impairing NK cell antitumor functions and impacting on NK cell migration and recruitment [32, 33]. In NK cells, TGF-β1 inactivates the serine and threonine kinase mTOR (mammalian target of rapamycin), reducing NK proliferation, metabolic activity and cytotoxic activity [32].

Here, we investigated how TGF-β1/TGF-βR1 signaling in PDAC cell and CAF contribute to induce decidual-like NKs and assessed the impact of its pharmacological inhibition on NK cell re-education.

## Material and methods

### Cell culture, maintenance, and treatments

Human, MIAPaCa2, BxPC3 and PANC-1, and murine FC1199 pancreatic cancer cell lines one human PDAC-CAF cell line, were used.

Primary PDAC-CAFs (pCAFs) were obtained from PDAC tumor tissue of a single patient.

Human umbilical vein endothelial cells (HUVEC, Lonza) were used for in vitro tube formation assay. Human K562 cells were used as target tumor cells, in the natural killer cell degranulation assay. Cell culture conditions are detailed in the section “Cell culture, maintenance, and treatments”, in Supplementary Materials and Methods.

MIAPaCa2, BxPC3 and PANC-1, PDAC-CAFs a pCAFs were treated with galunisertib (10 µM) and used to generate conditioned media (CM) (Supplementary Materials and Methods*)*.

### Clinical samples from PDAC patients

20 mL of whole blood in EDTA and fresh tissue samples (at upfront surgery) were collected from PDAC patients and processed within 1 h from collection. Patients with HIV, HCV and HBV infections, diabetes, or treated with chemotherapy or radiotherapy were excluded from the study. Patients (clinical features in Supplementary Table 1) were recruited at IRCCS MultiMedica, Milan, Italy within May 2022 and December 2023, and at IRCCS Istituto Clinico HUMANITAS, within October 2024 and June 2025.

### Polarization of human PBMCs in vitro

Total PBMCs (3 × 10^6^ cells/ml) isolated from HCs were cultured with 50% (v/v) of CM of MiaPaCa2, BxPC3, PANC-1, CAF, pCAF cells, treated or not with GAL (10 μM) in RPMI 1640 supplemented with 10% FBS, 2 mM L-glutamine, 100 U/L Penicillin and 100 mg/ml Streptomycin. TGF-β1 (10 ng/mL) was used as positive control. PBMCs were polarized for 72 h, pulsed with treatments at day 0 and at 48 h, then analyzed by flow cytometry. NK cells from HC were also polarized with HGF (25 ng/mL), IL-11 (100 ng/mL), IL17a (100 ng/mL), VEGFa (100 ng/mL), bFGF (50 ng/mL) and analyzed by flow cytometry.

The effects of CM on NK cell polarization and degranulation capabilities were assessed by multicolor flow cytometry.

### Flow cytometry

Multicolor flow cytometry analysis was performed on human total PBMCs and tumor-infiltrating mononuclear cells (MNCs) from PDAC patients and controls, on human total PBMCs polarized with CMs and on human total PBMCs and tumor-infiltrating MNCs from mice challenged with FC1199 cells, treated with galuniserib or vehicle. Degranulation assay was performed by flow cytometry, by detecting CD107a levels, on NK cells pre-treated with CM, and co-cultured with K562 cells. For staining procedures, see section “Flow Cytometry”, in Supplementary Materials and Methods. Gating strategies are shown in Supplementary Fig. 1A, B. Antibodies used in the study are listed in Supplementary Table 2.

### Characterization of conditioned media

CM from PDAC and CAFs cell lines, treated or not with GAL, and CMs from circulating CD9^+^ and CD9^-^ NK cells from patients, were characterized using the Human Cytokine Array C1000 (RayBiotec) (See section “Characterization of conditioned media” in Supplementary Materials and Methods).

### RNA extraction and real-time PCR

Real-Time PCR analysis was performed on liquid nitrogen frozen murine tumor fragments and on magnetically cell-sorted NK cells isolated from pancreas of tumor-bearing and controls mice using the SYBR Green Master Mix on the QuantStudio 6 Flex Real-Time PCR System Software. All reactions were performed in triplicate. The β-actin gene was used as housekeeping gene and results were showed as 2^−ΔΔCt^. (See section “RNA extraction and Real-Time PCR” in Supplementary Materials and Methods).

### In vivo experiments

5 × 10^4^ FC1199 PDAC cells were orthotopically transplanted in the pancreas in 10-week-old female C57BL/6J mice (Envigo, Correzzana, Italy). Non-tumor-bearing control mice underwent the same surgical procedures (sham control mice) to consider the potential inflammatory response due to surgical procedures.

Tumor growth was evaluated by weekly abdominal palpation. Mice were weighted every other day as a measure of drug toxicity. No adverse events were reported.

GAL treatments started 11 days after tumor injection. GAL was administered orally, every day at the dose of 75 mg/kg twice/day. Control group received the same volume of vehicle. Animals were euthanized 23 days after tumor injection and pancreas was used for NK isolation and IHC. Peripheral blood and tissue samples were used for multicolor flow cytometry and IHC analysis (See sections “Flow Cytometry” and “Microscopy analysis”, in Supplementary Materials and Methods).

The medicinal leeches (*Hirudo verbana*, Annelida) (ILFARM SRL, Italy) were kept in lightly salted water (NaCl 1.5 g/L), at 20 °C, in aerated tanks. Leeches were injected at the level of the 20th metamere, with 40 μl of CMs of FACS-sorted circulating CD9^+^ and CD9^-^ NK cells, from PDAC patients. A total of 6 leeches have been used, 1 for each CM. The injected body wall region was dissected after 6 h. Treated animals were anesthetized by immersion in a 10% ethanol solution until they appeared completely asleep, before being injected and dissected. Leeches tissue samples were processed by optical and transmission electron microscopy (TEM) analysis, CD31 immunofluorescence, as detailed in the section “Microscopy Analysis”, in Supplementary Material and Methods.

### Dataset interrogation

Single cell RNA-Seq public gene expression data were obtained from the Gene Expression Omnibus (GEO), repository (https://www.ncbi.nlm.nih.gov/gds): GSE205013 and re-analyzed using the online open-source tool for single-cell RNA sequencing data Cellenics® (https://www.biomage.net/).

For data acquisition and preprocessing, a total of 8 independent datasets comprising transcriptomic profiles of Pancreatic Ductal Adenocarcinoma (PDAC) patients were included in this study. Seven datasets (GSE183795, GSE62452, GSE57495, GSE85916, GSE71729, GSE21501, and GSE79668) were retrieved from the Gene Expression Omnibus (GEO) using the GEOquery R package [34]. RNA-sequencing data from The Cancer Genome Atlas (TCGA-PAAD) were downloaded using the TCGABiolinks package [35]. Sample selection for the TCGA cohort was performed following the criteria described by Peran et al. [36], excluding samples with low tumor purity or inconsistent histology.

For data integration and batch effect correction, microarray and RNA-seq datasets were merged into a single expression matrix, retaining only common genes across all platforms. To address non-biological technical variations arising from different experimental protocols and platforms, the ComBat method was applied [37]. The efficacy of batch correction was assessed using Principal Component Analysis before and after adjustment. Clinical survival data (Overall Survival, OS) were harmonized by converting all time intervals to months (Supplementary Fig. 1C). Patients were stratified into “High” and “Low” expression groups based on the median expression value of the target gene within the integrated cohort. Survival probabilities were estimated using the Kaplan–Meier method, and differences between groups were evaluated using the log-rank test. To quantify the association between gene expression and patient survival, Hazard Ratios (HR) with 95% Confidence Intervals (CI) were calculated using univariate Cox proportional hazards regression models. Differential expression analysis between High and Low expression groups was performed using the limma R package [38]. Differentially expressed genes (DEGs) between *TGFB1*^high^Vs*TGFB1*^low^ and *TGFBR1*^high^Vs*TGFBR1*^low^ are listed as Supplementary Tables 4, 5.

### Statistical analysis

Statistical analysis was performed using GraphPad Prism software v10 (GraphPad Prism Inc., San Diego, CA, USA). The normality of data distribution was assessed using the Shapiro–Wilk test. Results are shown as mean ± SD for normally distributed variables. On the contrary, data that didn’t pass the normality test, are shown as median with Interquartile range. Statistical difference between two datasets was determined using two-tailed Student’s *t* test, while for multiple datasets one-way ANOVA test was used. *P* values (*p*) ≤0.05 were considered statistically significant. Parametric tests were used to analyze data that satisfied the assumption of normality, otherwise, non-parametric tests were used.

FACS data were acquired and compensated during acquisition with the BD FACS-Diva software, then exported as FCS 3.1 files and analyzed with FlowJo, version 10.8.0.

## Results

### Patients with PDAC are characterized by expansion of tissue-infiltrating decidual-like NK cells

**Fig. 1:**
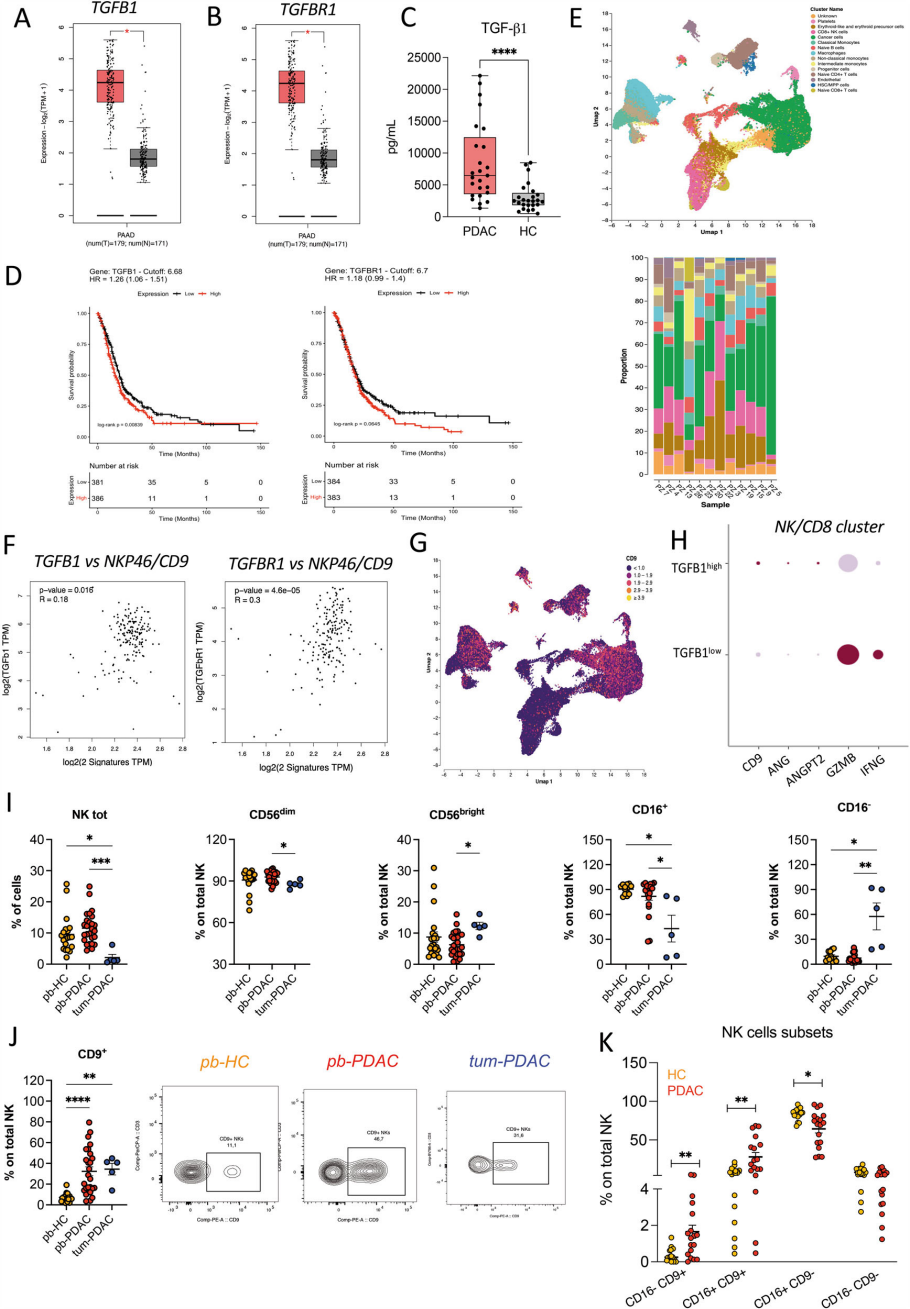
Identification of decidual-like NK cells in PDAC patients. TGF-β1 and TGF-βR1 expression in PDAC patients and healthy controls. **A** Box plots showing the expressions levels of TGF-β1 in PDAC patients (*n* = 179) vs normal subjects (*n* = 171) using data from the TCGA database in GEPIA2. **B** Box plots showing the expression levels of TGF-βR1 in PDAC patients (*n* = 179) vs normal subjects (*n* = 171) using data from the TCGA database in GEPIA2. **C** Plasma levels of TGF-β1 in 25 patients with PDAC compared with 25 healthy controls. TGF-β1 and TGF-βR1 expression level impact on PDAC patients’ survival: **D** Kaplan–Meier analysis showing the overall survival of PDAC patients dichotomized according to the median expression of TGF-β1 or TGF-βR1. Survival analysis was performed combining 8 different datasets (GSE183795, GSE62452, GSE57495, GSE85916, GSE71729, GSE21501, GSE79668 and PAAD-TCGA). Single-cell transcriptomics analysis of publicity available dataset: **E** scRNA-Seq analysis performed on 12 pancreatic samples from GSE205013; re-analysis of scRNA-Seq data from [40] using the Cellenics® open-source tool. UMAP plots show the different immune cell subpopulation and relative bar plots with the proportions of the diverse cell types in each PDAC patient. Color-coded by immune cell type. **F** Correlation analysis showing the positive association between TGF-β1 or TGF-βR1 and NKp46/CD9 signature. Correlation analysis was performed by GEPIA2 online tool based on TCGA project. **G** UMAP plots showing the expression of CD9 in each cluster of scRNA-seq data from ref. [40] using Cellenics open-source tool. **H** PDAC patients used in the scRNA-Seq were divided according to TGF-β1 expression. The expression of selected genes, namely CD9, ANG (angiogenin), ANGPT2 (angiopoietin-2), GZMB (Granzyme B), IFNG (Interferone-gamma) were assessed in the two groups in the NK/CD8 cluster. NK cell phenotyping in a cohort of PDAC patients and healthy controls: **I** Immunophenotyping of circulating NK cells from healthy controls (pb-HC; *n* = 12–20), PDAC patients (pb-PDAC; *n* = 20–26), and PDAC-tumor infiltrating NK cells (tum-PDAC; *n* = 5), for CD56, CD16 and **J** CD9 surface antigen expression by multicolor flow cytometry. Representative dot-plots for CD9, as decidual-like marker on NK cells are shown for pb-HC, pb-PDAC and tum-PDAC. Results are shown as Median with Interquartile range, One-way ANOVA followed by Kruskal–Wallis test: *p < 0.05, **p < 0.01, ****p < 0.0001. **K** Circulating NK cell subsets resulting from the combination of CD9 and CD16 surface antigen markers. (pb-HC; *n* = 15), PDAC patients (pb-PDAC; *n* = 18). Mann–Whitney Student’s *t* test: *p < 0.05, **p < 0.01.

We interrogated publicly available dataset for expression levels of TGF-β1 and TGF-βR1 in 179 human pancreatic cancers (T) compared to 171 normal controls (N). By GEPIA2 tool [39], we observed TGF-β1 and TGF-βR1 overexpression in pancreatic cancers compared to pancreatic normal tissues (Fig. 1A, B). TGF-β1 levels were confirmed to be significantly higher in the plasma of our cohort PDAC patients, compared with HC (Fig. 1C). Kaplan–Meier curves revealed that high levels of *TGFΒ1* in PDAC tissues, significantly correlate with reduced overall survival (*p* = 0.00839) (Fig. 1D). High expression of TGFBR1 was also associated to a poor prognosis, with a trend toward decreased overall survival (*p* = 0.0645). We used the Cellenics® open-source tool for single-cell RNA sequencing data, to dissect the immune landscape of 12 PDAC samples (Fig. 1E) from GSE205013 [40]. We initially investigated the TCGA dataset and found a significant positive correlation between both *TGF-β1/TGF-βR1* expression levels and signatures identifying decidual-like NK cells, namely *NKP46/CD9*, with NKP46 representing an NK-specific receptor and *CD9* a distinctive marker expressed by dNKs, respectively (Fig. 1F). We assessed the expression of the selected markers in patients with PDAC, with an in-silico approach. Sc-RNA-Seq data [40] showed that CD9 is expressed in PDAC samples not only by cancer cells, but also by some immune cells, also belonging to CD8/NK cluster (Fig. 1G). Clustering patients as *TGFβ1*^high^ and *TGFβ1*^low^ and assessing the expression of specific genes characterizing decidual-like NK cells, we found that *CD9*, *Angiogenin (ANG)* and *Angiopoietin 2 (ANGPT2)* genes resulted highly expressed by CD8/NK cluster of *TGFβ1*^high^ patients, while an increased expression of cytotoxic molecules, identifying activated NK cells were observed in *TGFβ1*^low^ patients (Fig. 1H).

To confirm these data, we assessed NK subset distribution in our cohort of PDAC patients, compared to a cohort of healthy controls (HCs). No significant differences in circulating total NKs, CD56^dim^ or CD56^bright^ NKs were observed between PDAC patients and HC (Fig. 1I). By contrast, CD16^-^ NKs were significantly enriched in the circulation and tumor tissues of PDAC patients (Fig. 1I). A statistically significant increased frequency of circulating and tissue-infiltrating CD9^+^ NK cells was found in patients with PDAC, compared with HC (Fig. 1J), with the expansion of both CD9^+^CD16^-^ and CD9^+^CD16^+^ circulating NK cells (Fig. 1K).

**Fig. 2:**
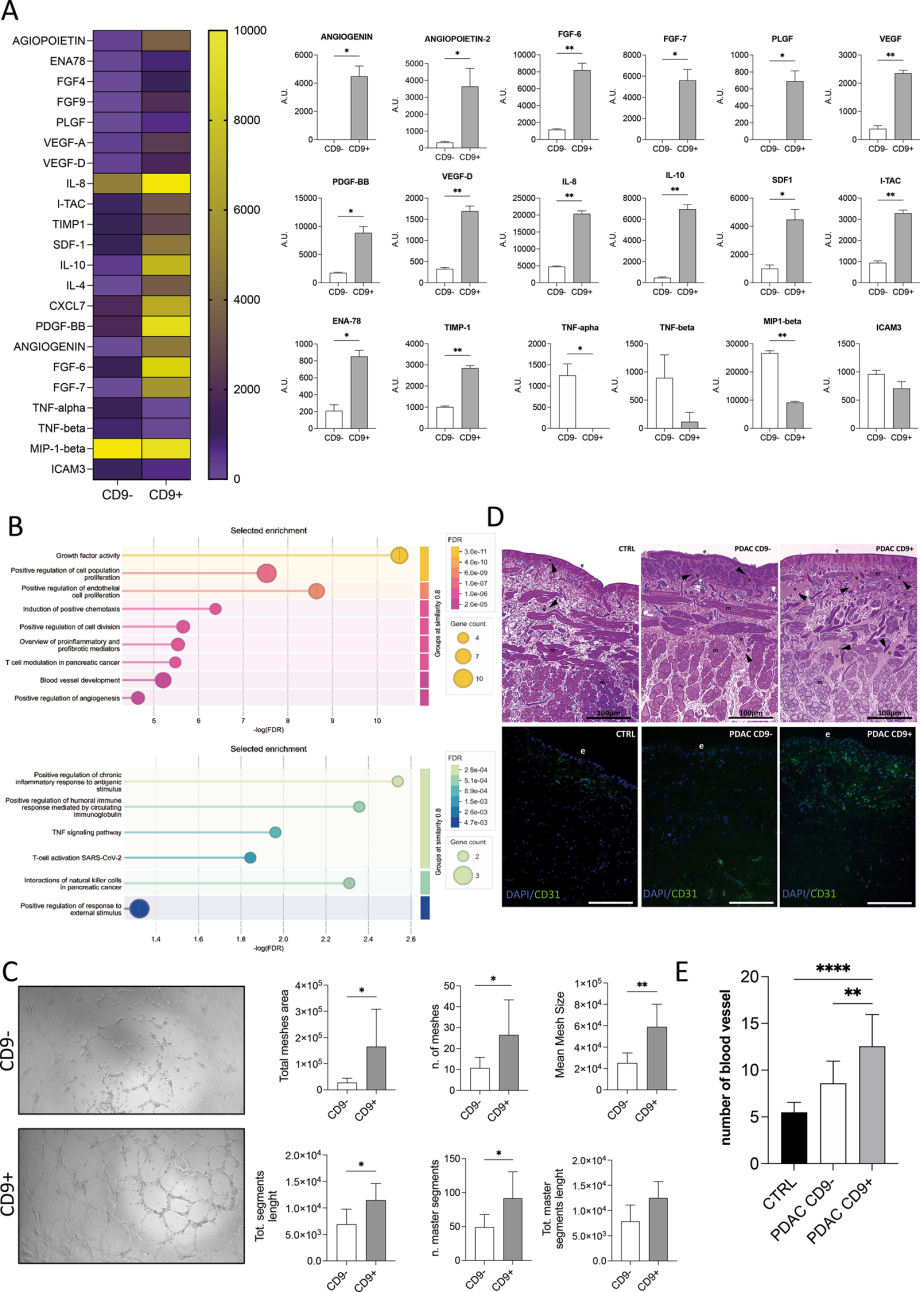
Functional characterization of CD9^+^ NK cells. CD9^+^ and CD9^-^ NK cell secretome profiling. **A** Secretomic analysis performed on conditioned media of FACS-sorted CD9^+^ and CD9^-^ NK cells, isolated from the peripheral blood of PDAC patients (pool of 3 different donors). Network and pathway analysis of factors released by NK cell decidual-like subsets: **B** STRING Network analysis of factors found upregulated and downregulated in CD9^+^ NK cells compared with CD9^-^ NKs. Pathway enrichment analysis was performed using Gene Ontology (GO), KEGG and WikiPathways databases to highlight the biological processes and signaling pathways most significantly associated with the upregulated (upper panel) and downregulated (panel below) proteins detected in the conditioned media of CD9^+^ NK cells vs CD9^-^ NK cells. In vitro characterization of pro-angiogenic effects of CD9^+^ NK cell-derived conditioned media: **C** Tube formation assay performed using HUVE cells exposed to CD9^+^ NK cell and CD9^-^ NK cell conditioned media for 6 h. Conditioned media from 3 different PDAC patients were used. Results are shown as mean ± SD. Unpaired Student’ *t* test: *p < 0.05. In vivo characterization of pro-angiogenic effects of CD9^+^ NK cell-derived conditioned media: **D** Crystal violet and basic fuchsin images and immunofluorescence (CD31, green; DAPI, blue) showing the induction of blood vessel by CD9^+^ NK conditioned media using the *Hirudo Verbana* model. CTRL: leeches injected with medium without FBS (negative control) (*n* = 3 leeches; 6 fields for animal); scale bars: 100 μm. **E** Quantification of blood vessels (number) in tissues from leeches injected with CM of CD9^+^ and CD9^-^ circulating PDAC NKs, or medium without FBS (negative control, CTRL).

We found that PDAC circulating CD9^+^ NK cells release higher amount of Angiogenin, Angiopoietin-2, FGF-6, FGF-7, PLGF, VEGFA, PDGF-BB, VEGF-D, IL-8, IL-10, SDF1, I-TAC, ENA-78, and TIMP-1 (Fig. 2A), compared with PDAC circulating CD9^-^ NK cells, factors involved in endothelial cell proliferation and blood vessel development, in chemotaxis regulation, in inflammation and positively regulating cell proliferation (Fig. 2B and Supplementary Fig. 2A, B). On the contrary, circulating CD9^-^ NK CM resulted enriched in TNFα, TNFβ, MIP1-β and ICAM3 (Fig. 2A) factors associated with a positive regulation of chronic inflammatory, humoral immune responses, and T-cell activation (Fig. 2B and Supplementary Fig. 2A, C).

To functionally evaluate the pro-angiogenic abilities of circulating PDAC-CD9^+^NK-CMs, we performed in vitro experiments. CMs from PDAC circulating CD9^+^ NKs significantly increased HUVEC tube formation, compared with CMs from PDAC circulating CD9^-^ NK CMs (Fig. 2C). Accordingly, CMs from circulating PDAC-CD9^+^NKs increased the number of blood vessel in *Hirudo verbana,* compared to CM from circulating PDAC-CD9^-^NKs and control medium without FBS (CTRL) Fig. 2D, E).

### Conditioned media from PDAC cell lines and PDAC-CAFs induce the decidual-like NK phenotype and limit NK cells antitumor activities

To test the ability of PDAC cell lines to generate dNK cells from circulating NKs of healthy donors, we used CM from MIAPaCa2, BxPC3 and PANC-1 PDAC cells, selected to reflect the molecular heterogeneity of PDAC [41], differences in immune evasion strategies and susceptibility to NK-mediated cytotoxicity [42, 43], and in CAF cross-talk and fibrotic signaling [44].

Besides these differences, using Protein Atlas online portal, we assessed that these cells were also characterized by a differential expression of TGFB1 and TGFBR1 genes (Supplementary Fig. 3A, B).

CM from a PDAC fibroblast cell line (CAF) and from primary CD90⁺EpCAM⁻CD45⁻ pCAF (Supplementary Fig. 3C), isolated in our lab from a patient with PDAC, was also used in NK cell polarization and functional assay. To confirm the ability of PDAC cell lines and CAFs to release TGF-β1, an ELISA assay was performed, showing that MiaPACA, BxPC3 and PANC-1 cells, as far as CAFs and pCAFs were able to secreted different levels of the selected marker (Supplementary Fig. 3D).

**Fig. 3:**
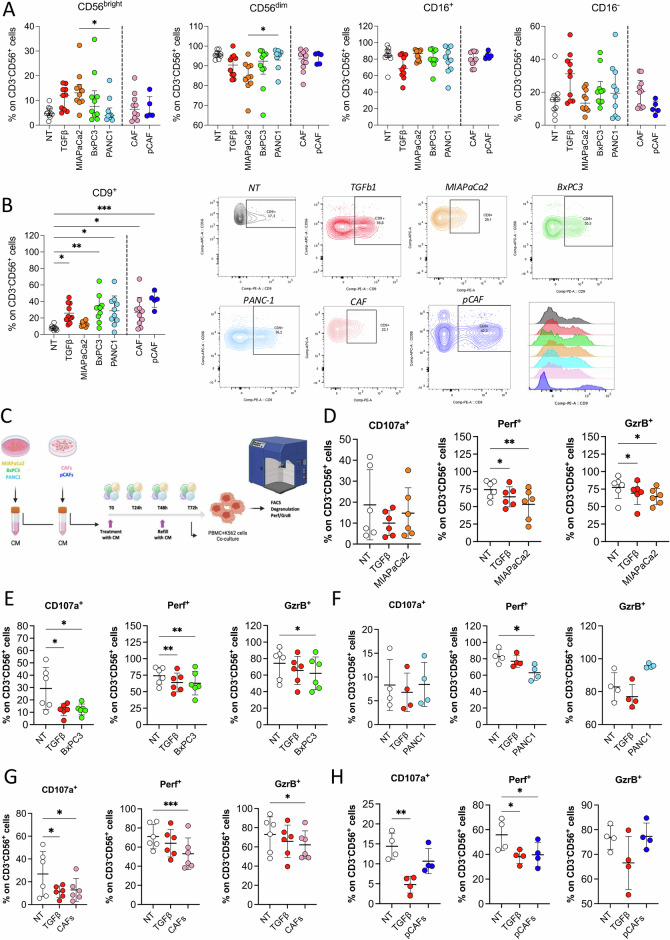
Effects of CM from PDAC cell lines and CAFs on NK cell polarization and antitumor activities. Conditioned media effects on NK cell phenotype. **A**, **B** Flow cytometry analysis for the effects of CM from PDAC cell lines (MIAPaCa2, BxPC3, PANC-1), a PDAC-CAF cell line (CAF) and primary PDAC-CAFs (pCAF) on the induction of decidual-like NK cells, based on CD56^bright^, CD56^dim^, CD16^+^, CD16^-^, CD9^+^ NK cell frequency. Representative dot plots and histograms for CD9, as the most upregulated decidual-like marker are showed in panel (**B**). *n* = 10 for NT, TGF-β1, MIAPaCa2, BxPC3, PANC-1 and *n* = 5 for pCAF. Results are shown as mean ± SD for CD9^+^ NK cells, One-way ANOVA; *p < 0.05, **p < 0.01 ***p < 0.001 and as Median with Interquartile range, Kruskal–Wallis test for CD56^bright^, CD56^dim^, CD16^+^, CD16^**-**^ NK cell, Kruskal–Wallis test: *p < 0.05. **C** Graphical representation of the degranulation assay schedule, following NK cell polarization with CM. Conditioned media effects on NK cell degranulation and activation: **D**–**H** Flow cytometry analysis for the effects of CM from PDAC cell lines (MIAPaCa2, BxPC3, PANC-1, *n* = 6 for each condition), a PDAC-CAF cell line (CAF, *n* = 6) and primary PDAC-CAFs (pCAF, *n* = 4) on NK cell degranulation (CD107a/Lamp1) and production of Perforin (Perf) and GranzymeB (GrzB). Results are shown as mean ± SD, One-way ANOVA; *p < 0.05, **p < 0.01 ***p < 0.001.

By exposing HC-derived NK cells to CM from the above-mentioned cell lines, we did not observe any change in the percentage of CD56^bright^ or CD56^dim^ NK cell subsets. The same trend was observed for CD16^+^ cytotoxic or CD16^-^ cytokine-producing NK cells (Fig. 3A). On the contrary, a statistically significant increase in CD9^+^ NK cells was found, upon NK exposure to TGF-β1 (10 ng/mL), or to BxPC3, PANC-1, CAFs and pCAF CMs (Fig. 3B).

We further evaluated the effect of CM on the production of cytotoxic molecules, by polarized NKs (Fig. 3C), including Granzyme B (GrzB) and Perforin-1 (Perf), along with NK cell degranulation abilities, assessed as CD107a production. We found that MIAPaCa2 cells significantly impaired the production Perf and GrzB, by NK cells, while no changes were observed in CD107a expression (Fig. 3D). CM from BxPC3 cell line negatively affected the production of CD107a, GrzB and Perf, by NK cells (Fig. 3E). PANC-1 CM stimulation was shown to reduce the frequency of Perf^+^ NK cells (Fig. 3F). A statistically significant decrease of CD107a and Perf was observed, following NK cell treatment with CAFs and pCAF CM, and CAF soluble factors were also able to negatively affect GrzB production, by NK cells (Fig. 3G, H).

### Galunisertib-treatment of PDAC cell lines and PDAC-CAFs limits the induction of decidual-like NKs and restore NK cell degranulation abilities

To assess the impact of GAL treatment on tumor PDAC cells and PDAC CAFs, we firstly evaluated its effect on cancer cell proliferation and invasion properties, also investigating GAL ability to modulate CAFs survival. We found that different concentrations of GAL did not affect MIAPaCa2, BxPC3, PANC-1 and CAF proliferation (Supplementary Fig. 3E), while we observed a trend toward reduced invasiveness in PDAC cell lines treated with GAL 10 μM for 48 h (Supplementary Fig. 3F).

**Fig. 4:**
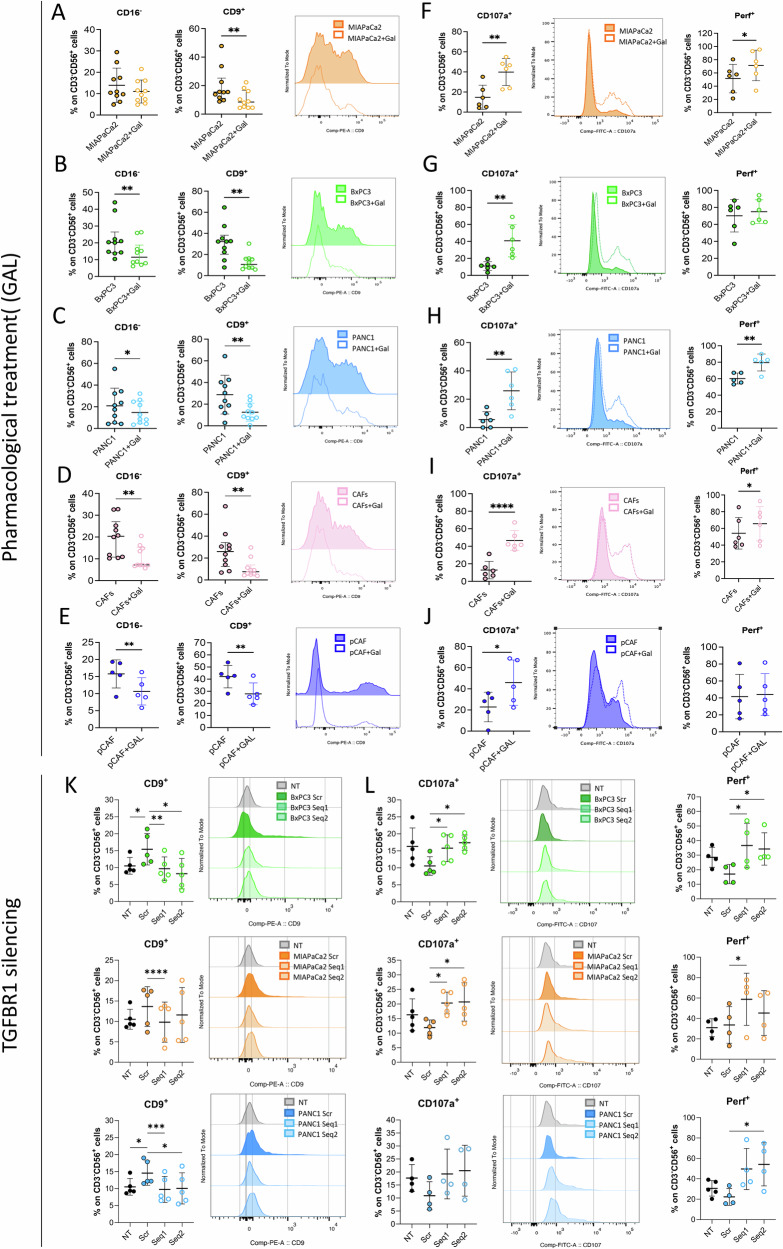
Effect of PDAC cells and PDAC-CAFs, treated with galunisertib, on NK cell polarization and activation status, via soluble factors. Flow cytometry analysis of the effects of conditioned medium (CM) from PDAC and PDAC-CAF on NK cell phenotype and cytotoxic abilities. The frequency of CD16^-^ and CD9^+^ NK cells was assessed following 72 h of stimulation with CM from GAL-treated or untreated. NK cell phenotype: **A** MIAPaCa2 (*n* = 10; Results are shown as mean ± SD for CD16^-^ NK cells and median with interquartile range for CD9^+^ NK cells, Paired *t*-test, **p < 0.01), **B** BxPC3 (*n* = 10; Results are shown as median with interquartile range, Wilcoxon test, **p < 0.01), **C** PANC-1 (*n* = 10, Results are shown as mean ± SD, Paired *t*-test, *p < 0.05, **p < 0.01), **D** CAFs (*n* = 10; Results are shown as median with interquartile range, Wilcoxon test, **p < 0.01), and **E** pCAFs (*n* = 5; Results are shown as mean ± SD, Paired *t*-test, *p < 0.05, **p < 0.01). Histograms displaying CD9 levels on NK cells stimulated with CM from GAL-treated or untreated cells are shown. NK cell degranulation and activation: Frequency of degranulating (CD107a^+^) and cytolytic (Perf^+^) NK cells following 72 h stimulation with CM from GAL^-^treated or untreated. **F** MIAPaCa2 (*n* = 6; Results are shown as mean ± SD, Paired *t*-test, *p < 0.05, **p < 0.01), **G** BxPC3 (*n* = 6; Results are shown as mean ± SD, Paired *t*-test, **p < 0.01), **H** PANC-1 (*n* = 6; Results are shown as mean ± SD, Paired *t*-test, **p < 0.01), (**I**) CAFs (*n* = 6; Results are shown as mean ± SD, Paired *t*-test, *p < 0.05, ****p < 0.0001), **J** pCAFs (*n* = 5; Results are shown as mean ± SD, Paired *t*-test, *p < 0.05), treated or not with GAL, were used. Histograms displaying CD107a levels on NK cells stimulated with CM from GAL-treated or untreated cells are shown. Effect of TGFBR1 silencing on NK cell phenotype and activation: **K** CD9^+^ NK cell frequencies and **L** CD107a^+^ and Perforin1 (Perf^+^) NK cells, upon stimulation for 72 h with CM derived from BxPC3 (green), MiaPACA-2 (orange) and PANC-1 (blue) either silenced (seq1, seq2) or expressing (Scr) TGFBR1. NT (non-treated) represents PBMCs cultured for 72 h in RPMI without CM and were used as baseline control. Results are expressed as mean ± SD, RM-one way ANOVA, *p < 0.05; **p < 0.001; ****p < 0.0001.

Thereafter, we treated PDAC cell lines and CAFs with GAL 10 μM and, following 48 h, CM from both treated or untreated cells were collected and used to polarize NK cells, isolated from healthy donors. We found that GAL treatment resulted in a trend toward decreases of CD16^-^ NK cells for CM from MIAPaCa2 cell line (Fig. 4A), that became statistically significant for NKs polarized with CM of BxPC3, PANC-1 cells, and CAFs (Fig. 4B–E), thus fostering the generation of CD16^+^ cytolytic NK cells (Supplementary Fig. 4A–E). Notably, GAL was effective in dampening the increase of CD9^+^ NK cell frequency, induced by the CM of PDAC cell lines and CAFs (Fig. 4A–E).

We also observed that treatment with GAL resulted in the generation of PDAC and CAF CM able to increase NK cell degranulation capabilities against K562 cells (Fig. 4F–J), also augmenting the production of Perforin, by NK cells (Fig. 4F–J and Supplementary Fig. 4F–J).

To confirm the relevance of TGFBR1/TGFB1 pathway on PDAC cells in promoting the generation of decidual-like/pro-tumoral NK cells, we transiently downregulated TGFBR1 in BxPC3, MiaPACA and PANC1 cells. We found an almost complete downregulation of *TGFBR1* following 72 h (Supplementary Fig. 5A), and the reduction in *TGFBR1* expression levels was also maintained after additionally 48 h, allowing CM generation, after siRNA wash-out (Supplementary Fig. 5B).

Functional assays using HC-derived PBMCs confirmed that TGFBR1 silencing was able generate CM capable to restrain the expansion of CD9^+^ NK cells, induced by PDAC cell CMs (Fig. 4K), also restoring NK-cell degranulation properties (Fig. 4L), as far as the production of Perforin (Fig. 4L) and Granzyme B (Supplementary Fig. 5C).

**Fig. 5:**
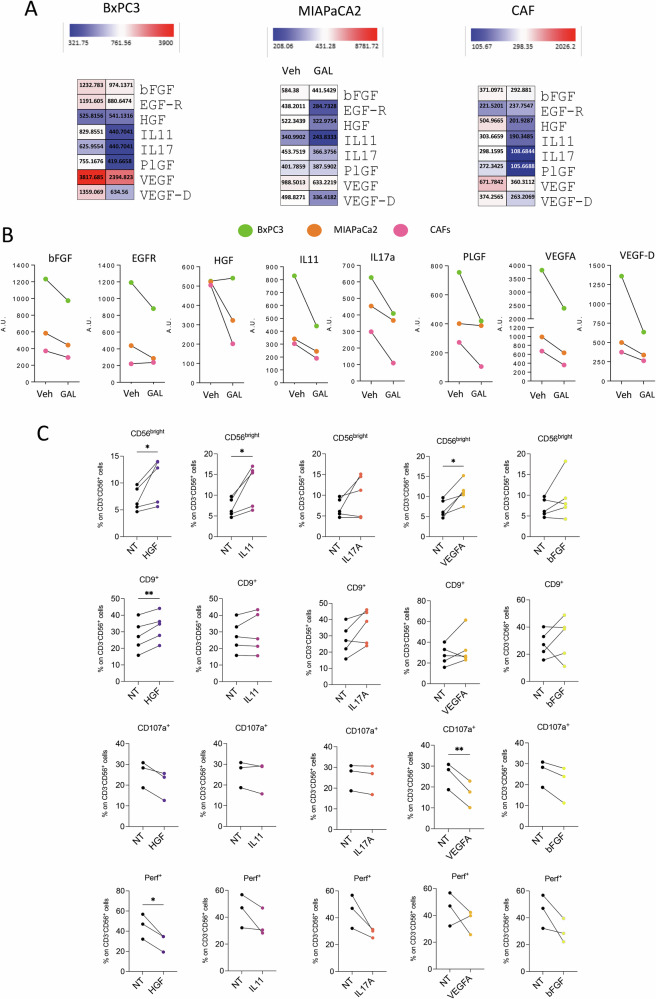
Identification of GAL-modulated soluble factors contributing to the polarization state of NK cells. **A** Secretome analysis of BxPC3, MIAPaCa and CAF cell lines treated with vehicle or GAL. **B** Selected soluble factors downregulated by GAL treatment in BxPC3, MIAPaCa and CAF conditioned media upon GAL treatment. Dots represent the mean of two biological replicate. **C** Phenotypic and functional modulations induced by HGF, IL11, IL17a, VEGFA, and bFGF on NK cells from healthy donors upon 72 h stimulation. Paired *t*-test, *p < 0.05, **p < 0.01.

Since the ability of tumor cells to induce pro-tumor polarization seemed to be independent from the levels of TGF-β1 released in their CM (Supplementary Fig. 3D), we explored the mechanisms through which GAL could restore the cytotoxic activity of NK cells, thereby restraining their pro-tumor polarization. We found that GAL was able to down-regulate different soluble factors, either in BxPC3 and MIAPaCa cells or CAFs (Fig. 5A, B; Supplementary Fig. 6A, B), including some molecules directly involved in the inhibition of NK activation, induction of fibrosis and angiogenesis, such as FGF2 (bFGF), IL11 and VEGF or VEGF-D. Soluble EGFR (sEGFR) was downregulated, by GAL, in both BxPC3 and MIAPaCa2 cells, while HGF release was dampened mainly in MIAPaCa2 and CAFs, and PLGF in BxPC3 and CAFs (Fig. 5A, B).

Based on these results, we polarized, for 72 h, HC-derived PBMCs with recombinant HGF, IL-11, IL17a, VEGFa, bFGF (Fig. 5C and Supplementary Fig. 6D). We observed that HGF was able to induce the expansion of CD56^bright^ and CD9^+^ NK cells, parallel to reducing the frequency of Perforin^+^ NK cells; a trend toward decrease of CD107a was also found upon stimulation. IL11 was effective in increasing the frequency of CD56^bright^ and reduce, even if in a non-statistically significant manner, the frequency of Perforin^+^ NK cells. No statistically significant modulations were observed in response to IL17a and bFGF, although a trend toward decrease was found for CD107a^+^ and Perforin^+^ NK cells. Finally, we observed a statistically significant expansion of CD56^bright^ and a reduction of CD107a^+^ NK cells by VEGFA.

Using an in-silico approach, we showed that all factors we found downregulated by GAL in vitro are highly expressed (at transcriptional level) in patients with PDAC (Supplementary Fig. 7A), with a statistically significant increase for *FGF2, HGF, IL11*, and *PLGF* and a trend toward increase for *EGFR* and *VEGF* comparing PDAC patients and controls. Moreover, when these factors were tested as a signature, we found that (*FGF2/EGFR/HGF/IL11/PLGF/VEGF)*^high^ patients showed a shorter disease-free survival than (*FGF2/EGFR/HGF/IL11/PLGF/VEGF)*^low^ patients (Supplementary Fig. 7B, C). A positive correlation was observed for FGF2/EGFR/HGF/IL11/PLGF/VEGF signature (6 signature) and NKP46/CD9 (2 signature) (Supplementary Fig. 7D).

### Galunisertib restrains the decidual-like polarization promoting the activation of NK and CD8^+^ T cells in an orthotopic mouse model of PDAC

**Fig. 6:**
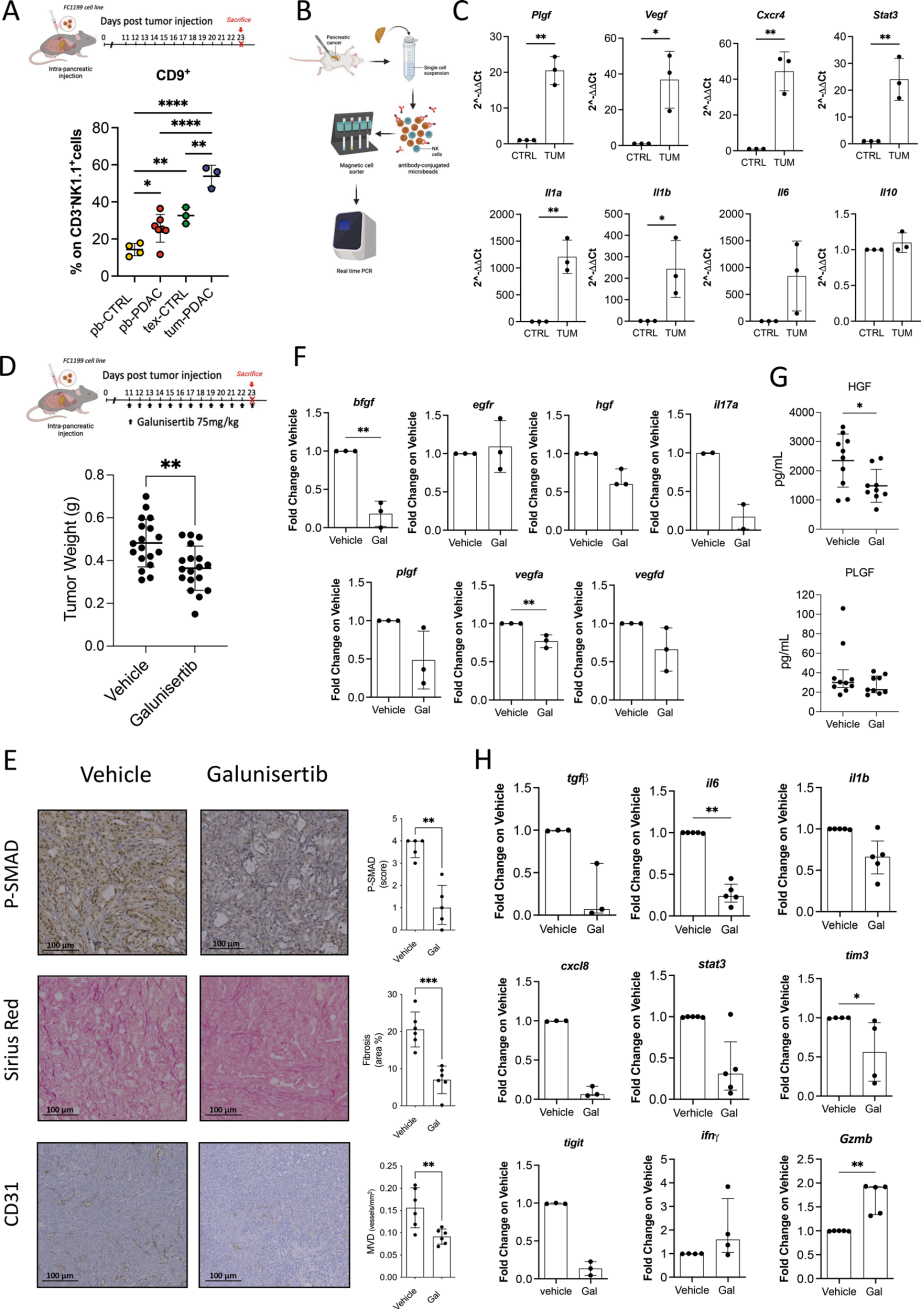
Galunisertib effect in shaping PDAC microenvironment. **A** Frequencies of decidual-like NK cells in immunocompetent naïve and tumor-bearing mice. Flow cytometry analysis for CD9^+^ (pb-CTRL *n* = 4, pb-PDAC *n* = 7, tex-CTRL *n* = 3, tum-PDAC *n* = 3). Results are shown as mean ± SD for CD9^+^ NK cells, One-way ANOVA; *p < 0.05, **p < 0.01, ***p < 0.001, ****p < 0.0001 and as median and interquartile range for CD49a+ NK cells, Kruskal–Wallis test: *p < 0.05. **B** Representative schedule reporting the steps of NK cell isolation from pancreas of control or FC1199-bearing mice and their molecular characterization is shown. **C** Molecular characterization of NK cells isolated from naïve and tumor-bearing mouse tissues: qPCR analysis for markers associated with the induction of decidual-like NKs, namely *plgf, vegf, cxcr4, stat3, il1-α, il-1β, il-6, il-10*, in NK cells isolated from pancreas of control or FC1199-bearing mice. Results are shown as mean ± SD, unpaired *t*-test; *p < 0.05, **p < 0.01, ***p < 0.001. **D** Representative treatment schedule for GAL administration in FC1199-bearing mice in vivo and effect on tumor growth (vehicle *n* = 18; GAL *n* = 18). Results are shown as mean ± SD, unpaired *t*-test; **p < 0.01. **E** IHC analysis for p-SMAD, collagen and CD31 in immunocompetent mice treated with vehicle or GAL: Effects of GAL on pSMAD (IHC), fibrosis (Sirius red staining) and microvascular density (CD31 staining) on excised pancreatic tumors from FC1199-bearing mice, treated or not with GAL. Representative field and quantification are shown. Results are shown as median and interquartile range for p-SMAD, Mann–Whitney test, **p < 0.01 and mean ± SD for Sirius Red and CD31, unpaired *t*-test; **p < 0.01, ***p < 0.001. **F** qPCR analysis of GAL-regulated genes in PDAC tumors: Effects of GAL on overall PDAC tumor bulk, as detected by qPCR, for genes that was found downregulated by GAL in in vitro experiments. Results are shown as median and interquartile range for *hgf* and mean ± SD, unpaired *t*-test for *bfgf, egfr, il17a, plgf, vegfa, vegfd*, unpaired *t*-test, **p < 0.01. **G** Plasmatic levels of HGF and PlGF of tumor-bearing mice treated with vehicle (*n* = 10) or galunisertib (GAL, *n* = 9) detected by milliplex, Results are expressed as mean ± SD for HGF, unpaired *t* test *p < 0.05 and as median and interquartile range for PlGF. **H** GAL effects in modulating pro-fibrotic and immune-related genes: Effects of GAL on overall PDAC tumor bulk, as detected by qPCR, for genes related to fibrosis (*tgfβ, il6*, *n* = 2–5), inflammation (*stat3, il1b, cxcl8*, *n* = 3–5) ad NK/CD8^+^ T cell activation (*Grzb, ifnγ, perf*, *n* = 4–5). Results are shown as median and interquartile range, Mann–Whitney test; *p < 0.05, **p < 0.01.

We found an expansion of circulating and tumor-infiltrating CD9^+^ NK cells, in mice orthotopically transplanted with FC1199 PDAC cells [45], compared to those detected in the peripheral blood and in normal pancreas of control not-injected sham mice (Fig. 6A). PDAC tumor-infiltrating NKs displayed statistically significant higher expression levels of *Plgf*, *Vegf, Cxcr4, Stat3, Il1a*, and *Il1b* and a trend toward increase was observed for *Il6*, compared to NKs from mice normal pancreas (Fig. 6B, C).

FC1199 PDAC tumor-bearing mice, treated with GAL, exhibited a statistically significant decreases in tumor weight (Fig. 6D), diaphragm invasion (only 5% of GAL-treated mice showed diaphragm invasion compared to 22% in the vehicle-treated group, data not shown), and ascites (15% of galunisertib-treated mice versus 28% of controls data not shown), together with reduced SMAD phosphorylation, decreased fibrosis and angiogenesis (Fig. 6E). These phenomena are associated with the reduction of the expression of pro-fibrotic factors and immunomodulatory cytokines both in the tumor bulk tissues and in plasma. In tumors, a statistically significant downregulation of *bfgf* and *vegf*, and a trend toward decrease for *hgf*, *il17a*, *plgf* and *vegfd* was observed in mice treated with GAL compared to vehicle-treated animals (Fig. 6F). HGF levels in plasma were significantly lower in GAL-treated mice, compared with control mice while PLGF levels, in GAL-treated tumors, were decreased but not significantly, corroborating results obtained in vitro (Fig. 6G). We also observed that GAL treatment induced a statistically significant downregulation of *Il6* and *Tim3*, in the tumor bulk, with a parallel increase of Granzyme B (Fig. 6H). A trend toward decreases of immunosuppressive molecules, such as *Tgfb*, *Il1b*, *cxcl8, Stat3*, and *Tigit*, with a concomitant increase of *Ifng* cytotoxic molecule was also observed, in GAL-treated mice (Fig. 6H).

**Fig. 7:**
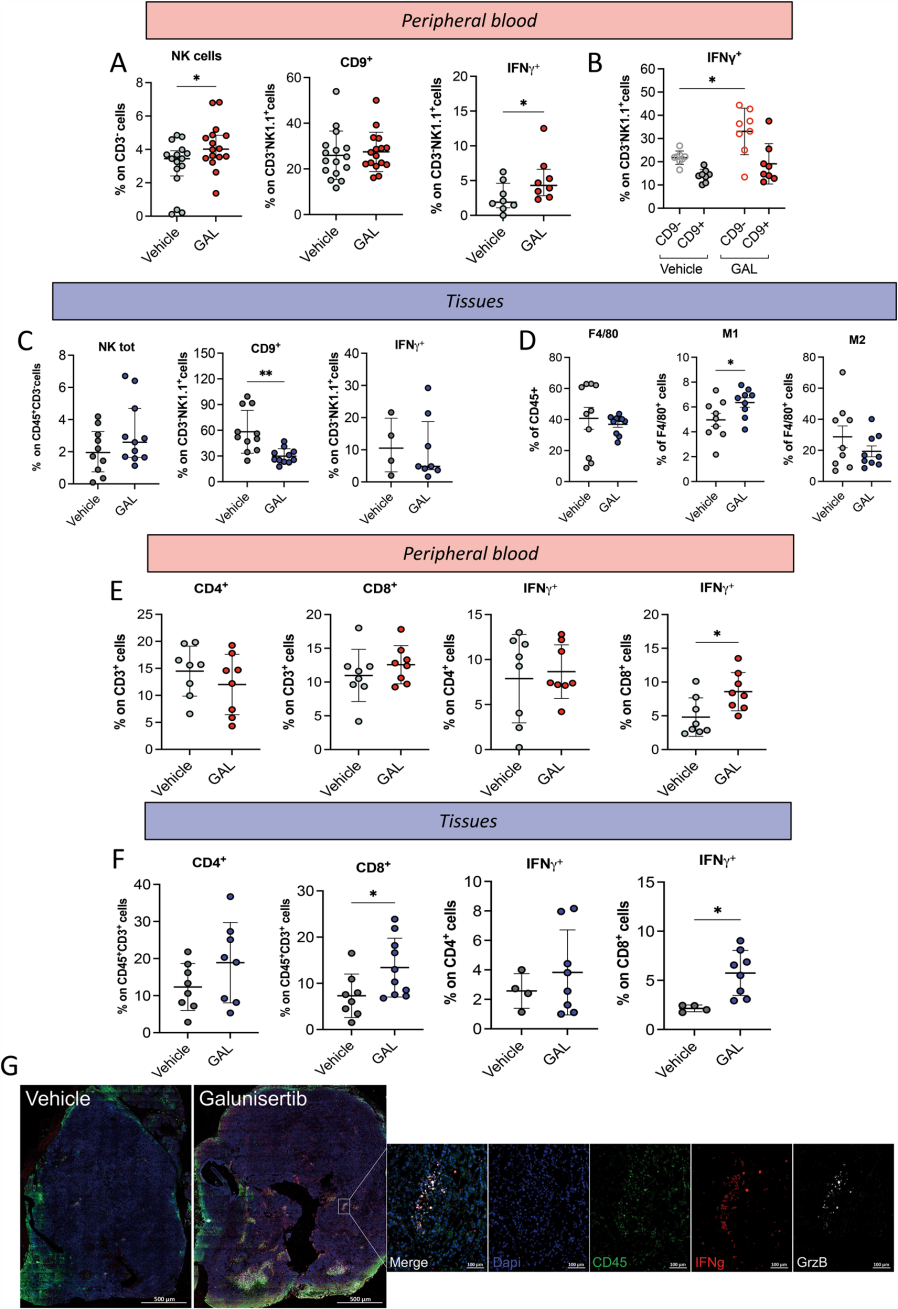
Effects of galunisertib on NK cells polarization, re-education and on PDAC-TME in vivo. **A** Effects of galunisertib on circulating NK cells polarization. Frequencies of total NK cells, CD9^+^ NK cells and IFNγ^+^ NK cells in the peripheral blood of mice injected with FC1199 and treated with vehicle or galunisertib (GAL) (*n* = 8–16); Results are shown as mean ± SD for CD9^+^ NK cells, unpaired *t*-test, *p < 0.05 and as median and interquartile range for total NK cells, and IFNγ^+^ NK cells, Mann–Whitney test, *p < 0.05. **B** Effects of galunisertib on circulating NK cells activation: Frequencies of IFNγ^+^ CD9^+^ and IFNγ^+^ CD9^-^ NK cells in the peripheral blood of mice injected with FC1199 and treated with vehicle or galunisertib (GAL) (*n* = 8). Results are shown as mean ± SD, unpaired *t*-test, *p < 0.05. **C** Effects of galunisertib on tissue-infilatrating NK cells polarization and activation: Frequencies of total NK cells, CD9^+^ NK cells and IFNγ^+^ NK cells within tumor tissue of mice injected with FC1199 and treated with vehicle or galunisertib (GAL) (*n* = 4–11); Results are shown as mean ± SD for CD9^+^ NK cells, unpaired *t*-test, **p < 0.01 and as median and interquartile range for total NK cells, and IFNγ^+^ NK cells. **D** Effects of galunisertib on macrophage polarization: Effects of GAL on F4/80 total macrophage infiltration (*n* = 10), and M1 (CD80, *n* = 9) and M2 (CD206, *n* = 9) polarization. Results are shown as median and interquartile range for F4/80 and as mean ± SD for M1 and M2, unpaired *t*-test, *p < 0.05. **E** Effects of galunisertib on circulating T-cells: Effects of GAL on circulating CD4^+^ and CD8^+^ T cells (*n* = 8–10), also evaluating their activation state in term of IFNγ production (*n* = 8–10). Results are shown as mean ± SD, unpaired *t*-test, *p < 0.05. **F** Effects of galunisertib on tissue-infiltrating T-cells: Effects of GAL on tissue-infiltrating CD4^+^ and CD8^+^ T cells (*n* = 8–10), and their activation state in term of IFNγ production (*n* = 4–8). Results are shown as mean ± SD, unpaired *t*-test, *p < 0.05. **G** Immunofluorescence analysis showing immune infiltration and effector activity in FC1199-bearing mice treated with galunisertib: Representative immunofluorescence analysis of tissue derived from FC1199-bearing mice treated with galunisertib or vehicle and stained for CD45 (green), IFNγ (red) Granzyme B (white) and DAPI (blue).

GAL-treated mice showed a statistically significant increase of circulating IFNγ^+^ NKs compared with control mice (Fig. 7A). Although no statistically significant changes were observed in the frequency of circulating CD9⁺ NK cells between tumor-bearing and control mice, analysis of IFN-γ production revealed that GAL was able to increase the proportion of CD9⁻ IFN-γ⁺ NK cells (Fig. 7B). A trend toward increases in the frequency of tumor infiltrating NKs, was observed, with a statistically significant decrease of CD9⁺ NK cells (Fig. 7C). Also, we observed an increase of antitumor M1 macrophages infiltrating cells in GAL-treated mice (Fig. 7D). GAL was able to promote the expansion of circulating IFNγ^+^ CD8^+^ T cells (Fig. 7E) and increased frequency of total and IFNγ^+^ producing CD8^+^ T cells within tumor tissues, compare with vehicle-treated mice (Fig. 7F). A representative immunofluorescence analysis performed on pancreas of mice injected with FC1199 and treated with vehicle or GAL confirmed the results obtained by flow cytometry, showing an increase of CD45^+^ cells in tumors of GAL-treated mice compared with vehicle (Fig. 7G). Accordingly, the re-activation of the immune landscape was further supported by the co-localization between CD45, GrzB and IFNγ only in GAL-treated mice (Fig. 7G).

## Discussion

Pancreatic ductal adenocarcinoma (PDAC) represents the third leading cause of cancer-related deaths worldwide [46]. It is associated with poor clinical outcomes, largely attributed to incomplete responses to standard therapies, the absence of reliable screening strategies and the lack of early diagnostic biomarkers. Multiple factors contribute to PDAC development and progression, including the activation of different oncogenes, aberrant metabolic reprogramming, and the presence of a desmoplastic and immunosuppressive microenvironment, characterized by the increase of different immunomodulatory cytokines and chemokines. Among these determinants, in the last years, TGF-β has emerged as a key mediator in PDAC pathogenesis [47]. We show that TGF-β1 is detected at higher levels in patients, than in healthy controls, either at RNA and protein level and its expression is associated with shorter survival. Although conflicting findings exist in the literature, regarding the association of TGF-β1 expression with survival in PDAC, mainly due to a limited sample size, our data are corroborated by different studies [48–50], showing that *TGFβ1*^high^ patients are characterized by a poor prognosis compared with *TGFβ1*^low^ patients, thus pointing out TGF-β1 as a possible biomarker for PDAC progression and outcome.

The tumor microenvironment (TME) in pancreatic ductal adenocarcinoma (PDAC) is dominated by immunosuppressive cells. Among the mechanisms that drive immunosuppression, elevated TGF-β1 levels and stroma-derived inhibitory signals have been recognised to play a crucial role in shaping PDAC TME [51], thus pointing out the relevance of TGF-β1/ TGF-βR1 axis. A previous study showed that tumor and stromal cell-derived TGF-β1 downregulates key activating receptors such as NKG2D on NK cells, contributing to impaired degranulation and reduced perforin/granzyme B release [52]. Moreover, tumor-derived extracellular vesicles enriched in TGF-β1 further suppress NK cell function by activating Smad signaling pathways [12]. In this context, we have previously observed that TGF-β1 acts as a major factor able to induce the decidual-like phenotype in NK cells, enhancing the expression of CD9 surface antigen on NKs, and promoting the expansion of pro-angiogenic circulating and tissue-infiltrating NK cells, concomitantly restraining their cytotoxic abilities [24–27, 53]. Here, we extend this knowledge by showing that NK cell polarization toward a tumor-permissive phenotype, marked by CD9 upregulation, is not only a direct effect of soluble TGF-β1 on NKs but also an indirect consequence of TGF-β1 signaling within tumor cells and CAFs.

Performing an in-silico analysis, we showed that CD9 expression is not only confined to cancer cells, or endothelial cells, but is also shared with the immune compartment, including tumor-infiltrating NK cells (CD8/NK cluster). Notably, clustering patients as TGF-β1^high^ and TGF-β1^low^, an enrichment of *CD9*, *angiogenin* and *angiopoietin-2* was observed in the CD8/NK cluster with a parallel reduction of *GZMB* and *IFNG*, suggesting a possible association between TGF-β1 levels and decidual-like NK cells in PDAC patients, as further suggested by a positive correlation between *TGFB1* or *TGFBR1* and decidual-like NK signature (*NKP46/CD9*). These results were corroborated in our cohort of PDAC patients, demonstrating an enrichment of both tissue-infiltrating and circulating CD9^+^ NK cells, highlighting that the use of a pharmacological inhibitor of TGF-β1/TGF-βR1 pathway can also result in the modulation of PDAC microenvironment in an NK-oriented fashion. Different studies are pointing out the relevance of NK cells in PDAC context, suggesting that the presence of activate NK cells within tumor tissue can impact on tumor progression [14, 15, 54]. By characterizing not only circulating NK cells but also uncovering the presence of circulating and tissue-infiltrating decidual-like NK cells, endowed with pro-angiogenic functions, both in vitro and ex vivo, our findings expand the current understanding of NK-cell heterogeneity in PDAC and open an entirely new avenue for immunological investigation, that will deserve further investigations.

To investigate the role of the TGF-β1/TGFβ-R1 axis in regulating NK cell phenotype/functions, we used the TGFβ-R1 inhibitor galunisertib (GAL) showing its ability in limiting the decidual-like polarization of NK cells promoted by PDAC tumor cells and CAFs, also restoring NK killing abilities.

Since the ability of tumor cells to induce a pro-tumor polarization was independent from the levels of TGF-β1 released in their CM, a secretome analysis was performed, showing that GAL treatment decreases the release of FGF2, sEGFR, VEGF, HGF, IL11, and PLGF, factors that are upregulated in PDAC patients compared with normal tissues and that can negatively impact on patient’s survival. Other groups have shown that high serum HGF correlate with pancreatic tumor progression [55–58]. Likewise, VEGF remains a relevant target in advanced PDAC; indeed, bevacizumab, a recombinant humanized monoclonal antibody that binds VEGFA, blocking its interaction with its receptors, has been shown to improve progression free survival, when combined with gemcitabine and erlotinib [59, 60]. Kim et al. have recently shown that chemotherapy prompts the production of PlGF and VEGF, activating CAFs and promoting ECM remodeling and fibrosis in PDAC, thereby affecting patients’ prognosis due to increased collagen deposition. The use of a novel drug (combining the single-chain Fv of atezolizumab (anti-PD-L1) to VEGF-Grab) to target PD-L1-expressing CAFs with chemotherapy, therefore limiting PLGF/VEGF-induced fibrosis, resulted in improved treatment outcomes [61]. The concept of reprogramming dysfunctional NK cells, by targeting TGF-β-mediated polarization, has been explored also in other tumors. In glioblastoma models, blocking the αv integrin/TGF-β axis enhances NK-mediated antitumor activity, particularly when combined with NK adoptive transfer [62]. Recently, Rea and colleagues demonstrated that SMAD4^KO^ NK cells were resistant to TGFβ inhibition, preserving effector functions, cytokine secretion showing an increased tumor penetration and tumor growth [63].

Our in vivo data demonstrating reduced CD9⁺ NK cell infiltration and the parallel increase of IFN-γ production are in line with these observations, also supporting the idea that the inhibition of the decidual-like NK conversion offers promising avenues to restore NK cytotoxic functions in tumors. Moreover, we also showed that galunisertib effects are not restricted to NK cells but can also improve CD8⁺ T cell infiltration and activation and promote M1 phenotype, suggesting that TGF-β pathway blockade can reprogram the overall TME toward antitumor immunity. Notably, consistent with literature reporting anti-fibrotic and anti-vascular effects upon TGF-β targeting [51, 64] we observed reductions in fibrosis and tumor angiogenesis. In this context, the alterations in the immune landscape with the reduction of tissue-infiltrating CD9^+^ decidual-like NK cells observed in treated mice, further sustain the impairment of angiogenesis. In vivo and in vitro data suggest that galunisertib, by simultaneously targeting immune cells, angiogenesis, and tumor cells may also exert a multifaceted anti-metastatic effect, reducing the spread of cancer to distant organs. Additional experiments are necessary to address the potential of TGFβ/TGFβR inhibitors to interfere with pancreatic cancer metastasis by acting both on tumor cells and TME.

We employed GAL based on its dual effects, namely reduction of fibrosis and immunosuppression, largely recognized as crucial hallmarks in PDAC.

We acknowledge that, despite strong pre-clinical evidence, GAL has failed to demonstrate a clear clinical benefit in unselected PDAC patient populations [65]. This highlights the complexity and heterogeneity of PDAC and suggests that inhibition of the TGF-β pathway alone may be insufficient when applied broadly [65]. Substantial quantity and timing variabilities in TGF-β/TGFBR1 expression in PDAC and cancer patients is observed, suggesting that only a subset of patients may harbor a TME that is permissive to TGF-β–targeted intervention. This clearly suggests that a more deep identification of TME/immune-oriented biomarkers, able in stratify PDAC patients, based on their TGFβ-/TGF-βR1 levels/scoring, would be beneficial to identify patients able to better respond to GAL, also based to their TME composition [66].

Although our data provide novel insights, limitations include the modest size of our patient cohort. Future directions should involve larger clinical studies to confirm the prevalence and prognostic significance of decidual-like CD9⁺ NK cells in PDAC and the correlation with clinical parameters. Furthermore, additional studies investigating the molecular mechanisms that underlay the pro-angiogenic switch of NK cells and the real contribution of this subset to PDAC progression are needed. Combinatorial strategies, including the association with checkpoint or chemotherapy, may yield synergistic enhancement of antitumor immunity, as suggested by Melisi and colleagues [67]. This concept is supported by other studies, including those from Mariathasan et al. [68] and Tauriello et al. [69], showing that, in colorectal cancer, TGFβ in the tumor microenvironment represents a key mechanism of immune evasion, inducing T-cell exclusion and inhibiting the acquisition of the TH1-effector phenotype. In this context, galunisertib treatment in tumor-bearing mice was sufficient to induce durable immunological memory, also improving the response to anti-PD-1–PD-L1 therapy [69].

These data raise the intriguing possibility that transient TGF-β blockade, when combined with PD-1/PD-L1 inhibitors, could unlock long-term antitumor immunity in tumors with immune exclusion, possibly re-shaping the immune landscape, restoring T-cell and NK cell cytotoxicity. Taken together, our study identifies a novel mechanism of TGF-β1-driven immune evasion in PDAC via tumor/CAF. Targeting TGF-βR1 with galunisertib not only restores NK functionality but also reshapes the TME by reducing pro-tumor secretome and stromal activation.

## Supplementary information

Original Data

Supplementary Material_FINAL

## Data Availability

The data supporting the findings of the study are available, by the corresponding authors, upon reasonable requests. In-silico analyses were performed using publicly available datasets. All datasets interrogated are reported in the “Dataset interrogation” paragraph of Material and Methods section.
