# Supplementary material for: The TGF-βR1 inhibitor galunisertib re-shapes the PDAC-TME by limiting decidual-like natural killer cells polarization
Source: Cell Death Dis. 2026 Mar 31;17(1):577. doi: 10.1038/s41419-026-08581-9 (PMC13276068; doi:10.1038/s41419-026-08581-9)

UNCROPPED WB/ARRAY

From Supplementary Figure 2

A

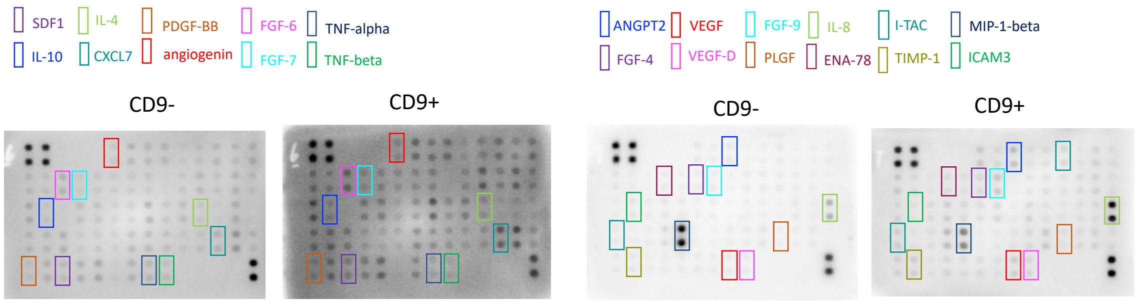

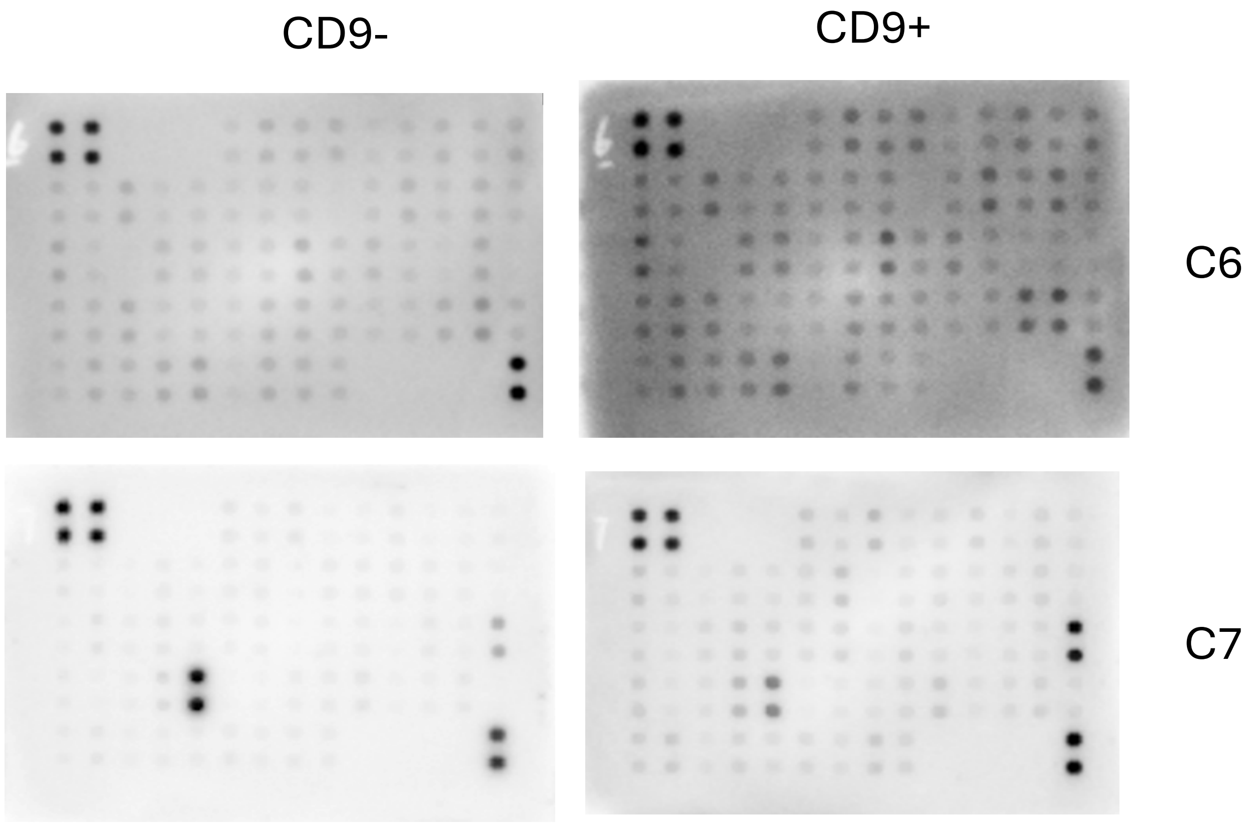

From Supplementary Figure 6

BxPC3 V

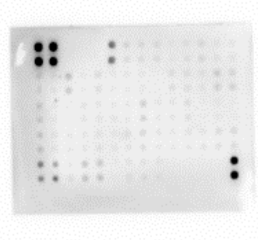

BxPC3 GAL

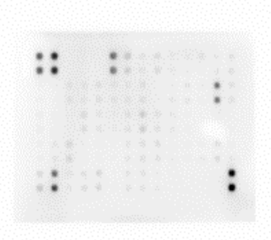

MIAPaCa2 V

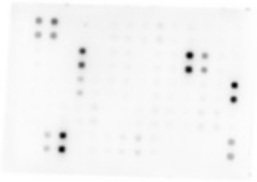

MIAPaCa2 GAL

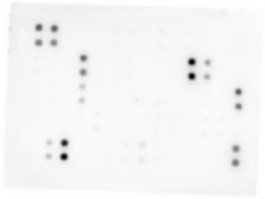

CAF V

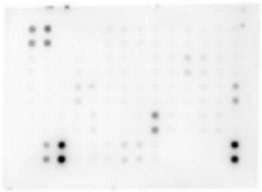

CAF GAL

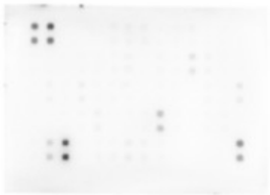

Supplement: Supplementary file 1 — Original Data [file 41419_2026_8581_MOESM1_ESM.pdf]
