# Supplementary material for: The TGF-βR1 inhibitor galunisertib re-shapes the PDAC-TME by limiting decidual-like natural killer cells polarization
Source: Cell Death Dis. 2026 Mar 31;17(1):577. doi: 10.1038/s41419-026-08581-9 (PMC13276068; doi:10.1038/s41419-026-08581-9)
Supplement: Supplementary file 2 — Supplementary Material_FINAL [file 41419_2026_8581_MOESM2_ESM.docx]

**SUPPLEMENTARY MATERIALS AND METHODS**

**Chemicals**

Galunisertib (GAL) (MedChem Express, Princeton, NJ, USA, HY-13226/CS-0474)) is a selective TGF-β receptor type I (TGF-βR1) kinase inhibitor. For *in vitro* studies, GAL was dissolved in DMSO at the concentration of 0,1M, stored at −80°C, and further diluted immediately before use. For *in vivo* studies, GAL was freshly dissolved in DMSO at the concentration of 0.3mg/μL and further diluted in corn oil, at the final concentration of 0.03mg/μL.

Cristal Violet (CV) was purchased by Sigma–Aldrich, Merck Life Science, Milano, Italy, (#C0775-25G).

**Cell culture, maintenance, and treatments**

The MIAPaCa2, human pancreatic cancer cell line were obtained from Istituto Zooprofilattico Sperimentale (Brescia, Italy); BxPC3 and PANC1 were obtained from American Type Culture Collection (ATCC). MIAPaCa2 and PANC1 were maintained in Dulbecco modified Eagle medium (DMEM) (Gibco, ThermoFisher, Rodano, Italy) supplemented with 10% FCS and 2mM L-glutamine. BxPC3 were cultured in RPMI 1640 (Euroclone) supplemented with 10% FBS, , 10mM Hepes, 1% NaPiruvate.

The FC1199 murine pancreatic cancer cell line was kindly provided by D.A. Tuveson (Cold Spring Harbor, NY, USA) and cultured in in Dulbecco modified Eagle medium (DMEM) (Gibco, ThermoFisher, Rodano, Italy) supplemented with 10% FCS and and 2mM L-glutamine.

PDAC-CAF were obtained from Vitro Biopharma (Neuromics, CAF08) and cultured in MSC-Gro™ media low serum maintaining medium (Vitro Biopharma, PC00B5).

Primary PDAC-CAFs (pCAFs) were obtained from PDAC tumor tissue of single patient, following mechanical processing, and maintained in DMEM (Euroclone) supplemented with 20% FBS, 2mM L-glutamine, 100 U/ml Penicillin and 100 mg/ml Streptomycin.

Human umbilical vein endothelial cells (HUVEC, #CC-2519 Lonza) were maintained in Vascular basal Medium (#PCS-100-030, ATCC) supplemented with Endothelial Cell Growth Kit-VEGF components (#PCS-100-041, ATCC), 100 U/ml penicillin and 100 μg/ml streptomycin (Euroclone). HUVECs were used between the three and five passages.

K562 cells (#CCL-243™ ATCC) were cultured in RPMI 1640 medium (Euroclone) supplemented with 10% FBS, 2mM L-glutamine, 100 U/ml Penicillin and 100 mg/ml Streptomycin and used for degranulation assay.

Cells were maintained at 37°C, 5% CO_2_ and were routinely tested for mycoplasma contamination (MycoBlue Mycoplasma Detector, #D101-01, Clinisciences, Italy).

**Crystal Violet assay**

Human MIAPaCa2, BxPC3 and PANC-1 PDAC cell lines, human PDAC-CAFs and primary human PDAC-CAFs were seeded in 96-well plate at 3x10^4^ cells/well. Following cell adhesion (24 h), cell media were replaced with fresh media containing 10% FBS and GAL, at increasing concentration of 0.1µM, 1µM, 10µM, 100µM. Cells were treated with GAL for 24-48-72 hours, then cells were fixed in 4% PFA and stained with crystal violet solution (Sigma–Aldrich, Merck Life Science, Milano, Italy, #C0775-25G). The staining was eluted with a 1:1 ethanol/0.1 M sodium citrate solution, and the absorbance was measured at 595 nm. Each condition was tested in triplicate.

**Transient transfection for TGFb-R1 silencing**

To achieve transient knock-down of TGFBR1 in human PDAC cell lines, 3x10^5^ BxPC3, and 2x10^5^  MiaPACA2 and PANC1 cells were seeded, in RPMI supplemented with 10% FBS and 1% Gln, in a six-well plate. Following 24h, cells were incubated for 72 h with a mix of RNAiMAX and siRNAs (s14071, seq 1; s14072, seq 2; s14073, seq 3, 20 μM) or Scramble negative control (Silencer Negative control, 20 μM, Thermo). *TGFBR1* expression levels were assessed using qPCR.

**Preparation of conditioned media (CM)**

PDAC cells and CAFs s were seeded 30,000/cm^2^ in complete medium for 24 h. Then, cells were washed and incubated with serum free medium ± GAL 10 µM. After 48h, CM were collected, centrifuged at 3000 rpm for 10 min at 4 °C, and stored frozen at -80 °C, until use. For CM from silenced or TGFBR1 expressing cells, following 72h treatment of BxPC3, MiaPACA2 and PANC1 cells with siRNAs or Scramble control, cell media were replaced (siRNA/Scramble wash out) and cells were cultured for additional 48h. *TGFBR1* expression levels were evaluated to confirm TGFBR1 knockdown maintenance.

FACS sorted CD9^+^ and CD9^-^ NK cells (50.000 NKs/50 μL) derived from peripheral blood samples of patients with PDAC, or healthy controls were seeded in 96-well plates in RPMI medium without FBS and cultured at 37 °C. Following 24 hours, conditioned media were collected, e centrifuged, transferred in a new tube and stored at -80 °C, until use.

**Characterization of conditioned media**

To identify the main soluble factors involved in microenvironment remodeling, immune-modulation and immune-cell recruitment and possibly modulated by GAL treatment, we characterized the conditioned media from PDAC cells (BxPC3 and MiaPACA-2) and CAFs using the RayBiotech C-Series Human Cytokine Antibody Array C1000 (AAH-CYT-1000), that allow the simultaneous detection of 120 different targets. Equal volume of CMs, derived from PDAC cell lines, PDAC-CAFs and primary PDAC CAFs, treated or not with GAL, were analyzed using the Human Cytokine Array C7 (#AAH-CYT-1000-8 RayBiotec) (Peachtree Corners, GA, USA), according to the manufacturer’s indications. Pool of 3 different patient-derived CMs, diluted in blocking buffer, were used for FACS sorted CD9^+^ and CD9^-^ NK cell secretomic analysis. All incubation and washes were performed under gentle rotation. After room temperature equilibration of reagents, membranes were blocked using Blocking solution and incubated 30 minutes at RT. After blocking solution removal, membranes were incubated with samples (1mL of total volume for each membrane) overnight at 4°C. The day after, samples were removed and membranes were washed with Wash buffer I (3 times, 5 minutes per wash RT) and Wash buffer II (twice, 5 minutes per wash RT). Biotinylated antibody cocktail was prepared following manufacturer instruction and incubated 2 hours at RT. Membranes were washed (as reported above) and then incubated with HRP-Streptavidin concentrate (diluted 1:1000 as indicated by manufacturer). Membranes were incubated 2 hours at RT, and then washed and chemiluminescent signals were detected mixing Detection buffer C and D (1:1 v/v) after 1-minute incubation. Chemiluminescent signal intensity was detected using the Alliance Q9 instrument (UVITEC). Optical density was determined using the ImageJ software and Dot plot Analyzer plugin.

**Isolation of PBMCs from human and murine whole blood**

Peripheral blood mononuclear cells (PBMCs) from human samples (PDAC patients and healthy controls (HCs)) were isolated from 20 ml of whole blood, collected in EDTA tubes. Whole blood samples were processed for platelet-depleted plasma isolation, using two serial centrifugations. Plasma was stored at -80°C until use. Blood was diluted with PBS 1:1 (v/v) and PBMCs were isolated by density gradient stratification with Ficoll Histopaque-1077 (#10771-500ML Sigma-Aldrich) at 2,000 rpm, 20 minutes, room temperature (RT). Human samples were used within 4 hours from collection, to avoid sample alteration possible modulating the detection of decidual-like NK cell frequency.

Murine blood samples were collected from tumor-free, control or GAL-treated mice by retromandibular vein sampling in 10% EDTA 0,5M. Plasma was separated by centrifugation at 3,200 g for 15 minutes at room temperature and red blood cells were lysed using ACK (Ammonium-Chloride-Potassium) solution (whole blood:ACK solution used 1:4 (v/v) ratio). Following centrifugation at 1,500 rpm, 5 minutes at RT, PBMCs were used for flow cytometry analysis.

**Cytometry**

For PBMCs derived from human samples (naïve or polarized) or mice, 2x10^5^ cells were stained with the following antibodies: PerCP-conjugated anti-CD3 (BW264/56) (130-113-131, Miltenyi Biotec), APC-conjugated anti-CD56 (REA196) (#130-113-310, Miltenyi Biotec), FITC-conjugated anti-CD16 (REA423) (#130-113-392, Miltenyi Biotec), PE-conjugated anti-CD9 (REA1071) (#130-118-807, Miltenyi Biotec), for human samples. PerCP-conjugated anti-CD3 (145-2C11) (#561089, BD Biosciences), BV650-conjugated anti- NK1.1 (PK136) (#564143, BD biosciences), BUV737-conjugated anti-CD9 (KMC8) (#741747, BD Biosciences), for murine samples. For tissue samples (both murine and human), single cells suspension was stained with Fixable/Viable die (#565388, BD Biosciences).

For surface antigen detection, cells were stained for 30 minutes at 4°C, at dark, with the appropriate antibodies. For intracellular staining, cells were stimulated with 4 h and following surface antigen staining, as above) cells were processed using the BD Cytofix/Cytoperm™ Fixation/Permeabilization Solution Kit (#554714, BD Bioscience), following the manufacturer’s indications. CD107a expression, as a readout for degranulation efficiency, was evaluated on CD3^-^CD56^+^ NK cells through multicolor flow cytometry. To determine NK degranulation efficiency, basal level of CD107a of NK cells alone was subtracted from NK cells co-cultured with K562 cells. For Perforin and Granzyme detection, following surface staining for CD3 and CD56, cells were fixed and permeabilized using Cytofix/Cytoperm kit (#554714, BD Bioscience), and then intracellularly stained with PE-CF594-conjugated Perforin (δG9) (#563763, BD Biosciences), PE-conjugated Granzyme B (GB11) (#761142, BD Biosciences) and BV650-conjugated IFNγ (4s.B3) (#563416, BD Biosciences).

NK cell degranulation ability was assessed by CD107a detection, following 72h polarization of PBMCs with CM from PDAC cell lines, CAFs and pCAFs, pre-treated or not with GAL. Following 72h-polarization, PBMCs (2x10^5^ cells) were co-cultured for 5 h with K562 (E:T ratio of 1:1), in presence of the FITC-conjugated anti-human CD107a/LAMP-1. PBMCs or K562 alone were used as internal controls. During the co-culture, cells were incubated for 4h with Golgi Stop and Golgi plug solutions (BD Bioscience), then used for flow cytometry analysis.

***In vivo* experiments**

Mice experiments.

For intra-pancreatic tumor injection, 10-week-old female C57BL/6J mice (Envigo, Correzzana, Italy) were anesthetized and left subcostal laparotomy performed. Spleen and pancreas were exposed, and the tumor cells suspension (5×10^4^ FC199 PDAC tumor cells) was injected in the pancreas. Organs were returned to the peritoneum, the abdominal wall closed with 4-0 vicryl surgical suture and skin sealed with surgical staples. Non-tumor bearing control mice used in the study underwent the same surgical procedures previously described without FC1119 injection (sham control mice) to consider the potential inflammatory response due to surgical procedures.

Two *in vivo* experiments were performed. In each experiment, mice were maintained under specific pathogen-free conditions and handled using aseptic procedures at Mario Negri Institute. The animals were randomized by body weight, prior to tumor injection and divided in groups of 5 (exp.1) or 4 (exp.2) mice. Then the groups of mice were allocated to control or treatment group. To reduce pain due to the surgical procedure, mice were treated with Carprofen 5mg/kg 2 hours before, 24 and 48 hours after surgery. Tumor growth was evaluated by weekly abdominal palpation. Mice were weighted every other day as a measure of drug toxicity. No adverse events were reported.

GAL treatments started 11 days after tumor injection. GAL was administered orally, every day at the dose of 75 mg/kg twice/day. Control group received the same volume of vehicle. Animals were euthanized 23 days after tumor injection and pancreas were used for NK isolation and IHC.

**Milliplex murine plasma profiling**

EDTA plasma samples, from mice, were processed within 30 min of blood drawing as described [27] and stored at -80°C, until use. Circulating levels of growth/angiogenic factors and cytokines were measured using the Luminex-based assay MAGPMAG-24K (Merck Life Science) and the BioRad Bio-Plex 200 Paltform.

***In vitro* tube formation assay**

14x10^3^ HUVECs /well were seeded in a 96 well plate, previously coated with 50 μL of 10 mg/mL Matrigel (354230, BD Biosciences). Plates were incubated at 37°C, 5% CO_2_, for 30 minutes, to allow for Matrigel polymerization. Endothelial cells were exposed to CMs (diluted 1:1 with medium without FBS) from FACS sorted CD9^+^ NK cell and CD9^-^ circulating NK cells from PDAC patients, and maintained for 6 hours, at 37 °C, 5% CO_2_. The ability of HUVEC to generate capillary-like structures (defined as tubes) was detected using a Zeiss Microscope, associated with a Nikon camera (Axio Observer A1, Zeiss). Tubulogenic efficiency was quantified using the Angiogenesis Analyzer tool, as package of the ImageJ software (U.S. National Institute of Health, Bethesda, MD, USA).

**Microscopy Analysis**

*Optical and transmission electron microscopy (TEM) on Leeches*

Injected areas of leech tissue were fixed in 4% glutaraldehyde diluted in 0.1 M cacodylate buffer (pH 7.4) for 2 hours. After several washings in cacodylate buffer, samples were post-fixed for 1h with 1% osmium tetroxide in cacodylate buffer. After washing, samples were dehydrated and embedded in an Epon-Araldite 812 mixture of epoxy resin (Sigma Aldrich, Italy). Sections were obtained with an RMC Power Tome XL (Boeckeler Instruments, USA).

Sections for light microscopy (700nm thickness) were stained with crystal violet and basic fuchsin and observed under the light microscope Nikon Eclipse (Eclipse Nikon, Japan). Data were recorded with a DS-5 M-L1 digital camera system (Nikon, Japan).

*CD31 Immunofluorescence on Hirudo verbana sections on Leeches*

Leech tissues were embedded into OCT (Tissue Tek® O.C.T. Compound, Electron Microscopy Sciences, Hatfield, USA, #62550-01) frozen in liquid nitrogen and stored at −80°C. Cryosections (7μm thickness) were obtained with a cryostat (Leica CM1850), collected on the gelatinated slides, and held at −20°C until use. For the immunofluorescent assays, slides were rehydrated for 10 minutes in phosphate buffer solution (PBS, NaCl 8 g/l; KCl 0.2 g/l; Na_2_HPO_4_ 1.44 g/l; pH 7.4) and incubated for 30 minutes in blocking solution containing 2% Bovine Serum Albumin (BSA) and 0.1% Tween, in PBS. The same solution was used to dilute primary and secondary antibodies. Samples were incubated at room temperature for 1 hour with mouse α-CD31 monoclonal antibody, diluted 1:200 (Novocastra, Nussloch, Germany, #NCL-CD31-1A10).

After several washes in PBS, samples were incubated with goat anti-mouse Cy3-conjugated secondary antibody (Jackson Immuno Research Laboratories, #115-025-146), diluted 1:200 at room temperature for 45 minutes. Nuclei were counterstained with DAPI (0.1 mg/ml 4,6-diamidino-2-phenylinedole, Sigma Aldrich, Italy, #D9542), diluted 1:5000 in PBS for 3 minutes and slides were mounted with Cityfluor (Electron Microscopy Sciences, Hatfield, USA, #17970-25). Negative control experiments were conducted, in which primary antibody was omitted.

Immunohistochemistry on murine tissue samples

Tumors were collected, fixed in 10% phosphate-buffered formalin, embedded in paraffin, and cut into 4 µm-thick sections. Sections were stained with Hematoxylin and Eosin (H&E) and Sirius red. For immunohistochemical analysis of pSMAD2/3, after antigen retrieval with Borg Decloaker RTU (Biocare Medical, Pacheco, CA, USA, BD1000 S-250) at 95°C for 90 minutes, anti-pSMAD2/3 rabbit monoclonal antibody was used (Cell Signaling, #8828),), followed by Rabbit-on-rodent HRP polymer (Biocare Medical, # RMR622 G ), and developed with DAB chromogenic kit (Biocare Medical, # DB801 L). For negative control no-primary antibody was used. Images (bright field) were acquired with Axio Imager Z2 (Zeiss, Felbach, Switzerland).

Tumor blood vessels were stained with rat monoclonal anti-mouse anti CD31 (PECAM-1), Clone SZ31 (Dianova, #DIA-310).

Presence of fibrosis was analyzed using ImageJ software (https://imagej.nih.gov/) and expressed as the percentage of positive area on total tumor area. The amount of pSMAD2/3 positive tissue in tumors was quantified by blind scoring, exploiting the difference in staining intensity in different areas. For microvascular density analysis, quantification of CD31^+^ vessels was performed using the free and open source Qupath software, quantifying three different fields for each slide.

Immunofluorescence on murine tissue samples

Tumors were collected from mice, fixed in 10% phosphate-buffered formalin, embedded in paraffin, and cut into 4 µm-thick sections. Antigen retrieval was induced by citrate buffer pH 6.0 (Thermo Fisher) at 90°C for 40 minutes. Sections were then exposed 4°C overnight to anti-IFNγ (1:100, goat polyclonal-ab, R&D Systems ,#AF-585-NA, anti-granzyme B (1:200, rabbit monoclonal-ab, Abcam, ,#ab255598) and anti-CD45 (1:100, Fitc rat monoclonal-ab, Purified Rat Anti-Mouse CD45, BD Pharmingen™, #553076); followed by secondary fluorescent antibodies (1:50 donkey anti-goat fitch (Jackson Immunoresearch, #705-095-003), 1:100 donkey anti-rabbit Cy5  (Jackson Immunoresearch, #711-**175**-152); donkey anti-rat Cy3( (Jackson Immunoresearch, #712-165-153). Images (fluorescence) were acquired with Axio Imager Z2 (Zeiss).

**RNA extraction and Real-Time PCR**

Total tumor RNA was extracted, from liquid nitrogen frozen murine tumor fragments, by homogenization with Ultra-Turrax (IKA, Thermo Fisher) in cold TRIzol (Invitrogen, Thermo Fisher, #15596018), purified by the miRNeasy Tissue/Cells Advanced Kits (#217684, Qiagen, Milano, Italy), and then reverse-transcribed with the High-Capacity cDNA Reverse Transcription Kit (Applied Biosystems, Monza, Italy, #4374966), according to the manufacturer’s instructions.

Total RNA from Trizol reagent (Sigma-Aldrich)-stored murine NK cells, isolated from pancreas of controls and tumor-bearing mice was extracted using the miRNeasy Mini Kit (QIAGEN, #217004) and its concentration was determined using a Nanodrop Spectrophotometer.

Genomic DNA was removed using DNase I Amplification Grade (Invitrogen, Thermo Fisher, #18068015), followed by reverse transcription of 500 ng of total RNA using the SuperScript VILO cDNA synthesis kit (Invitrogen, Thermo Fisher, #11754050).

Real-time PCR was conducted using the SYBR^TM^ Green Master Mix (#4309155 Thermo Fisher) on the QuantStudio 6 Flex Real-Time PCR System Software (Applied Biosystems, Thermo Fisher Scientific, USA). SYBR^TM^ Green Master Mix contains SYBR Green I dye, AmpliTaq Gold^TM^ DNA polymerase (hot-start enzyme), dNTPs, MgCl_2_, buffer and ROX reference dye. All reactions were performed in triplicate. The β-actin gene was used as housekeeping and results were showed as 2^^−ΔΔCt^. Primers sequences are provided in **Supplementary Table 3**.

**Invasion assays**

Tumor cell invasion was evaluated using modified Boyden chambers and 8 μm pore size PVP-free polycarbonate microporous membranes coated 1 mg/ml Cultrex Reduced Growth Factor (R&D, #3433-010-0). 5x10^4^ PDAC cells either silenced or expressing TGFBR1 were resuspended in 200 μL of serum-free DMEM and placed onto the upper chamber of a 24-well transwell plate. 750 μL of high glucose DMEM containing 10 % FBS was added into the lower transwell chamber. Following an O/N at 37°C and 5% CO2, top chambers, were removed, and invaded cells were fixed and stained with Hoecst (10 μg/mL). 5 randomly selected fields per well were counted using a fluorescence microscope (Leica). The number of fluorescent cells was assessed using ImageJ software.

**Bioinformatic analyses: Data Acquisition and Preprocessing**

Microarray probes were mapped to HGNC symbols, while for genes represented by multiple probes, expression values were aggregated by calculating the mean expression. Only samples annotated as primary tumors were retained, while normal or adjacent tissue samples were excluded based on clinical metadata. RNA-seq raw count data were converted to gene symbols and subsequently normalized using Variance Stabilizing Transformation to ensure homoscedasticity for downstream integration.

**Supplementary Figures and Tables**

**Supplementary Figure 1**

**Supplementary Figure 1: Gating strategies for flow cytometry (A)** Gating strategy used for NK cell phenotype analysis by flow cytometry, on human PBMCs polarized with CM. **(B)** Gating strategy used to detect NK cell degranulation (CD107a), Perforin (Perf) and GrazymeB (GrzB) production, on human PBMCs polarized with CM. (C). Principal Component Analysis before and after adjustment showing the efficacy of batch correction following ComBat method.

**Supplementary Figure 2**

**Supplementary Figure 2: Secretomic analysis of CD9^+^ and CD9^-^ conditioned media. (A)** Membrane array quantified for secretomic analysis. Targets of interested are displayed and highlighted with different colors. (**B**) STRING Network analysis of factors found up-regulated in CD9^+^ NK cells compared with CD9- NKs. Pathway enrichment analysis was performed using Gene Ontology (GO), and WikiPathways databases to highlight the biological processes and signaling pathways most significantly associated with the selected factors (**C**) STRING Network analysis of factors found up-regulated in CD9^-^ NK cells compared with CD9^+^ NKs. Pathway enrichment analysis was performed using Gene Ontology (GO), KEGG and WikiPathways databases to highlight the biological processes and signaling pathways most significantly associated with the selected factors

**Supplementary Figure 3**

**Supplementary Figure 3: Effects of GAL on PDAC and PDAC-CAFs**. Analysis for TGF-β1 **(A)** and TGF-βR1 **(B)** expression in different PDAC cell lines, including those used in the manuscript performed using the Human Protein Atlas program (https://www.proteinatlas.org). **(C)** Phenotypic characterization of pCAFs isolated from the tissue of a PDAC patient by flow cytometry. Representative histograms for CD90, CD45 and EpCAM are shown. **(D)** Detection of TGF-β1 production by MIAPaCa2, BxPC3, PANC-1 and PDAC-CAF cell line and primary PDAC-CAF (pCAF), as detected by ELISA assay. **(E)** Effects of Galunisertib on PDAC (MIAPaCa2, BxPC3, PANC-1) and PDAC-CAF cell line and primary PDAC-CAFs (pCAF) viability by crystal Violet assay. **(F)** Effect of 48h GAL treatment (10 μM) on MIAPaCa2, BxPC3, PANC-1 invasive properties. Results are shown as mean ± SD

**Supplementary Figure 4**

**Supplementary Figure 4: Effect of conditioned media (CM) derived from PDAC cells and PDAC-CAFs on NK cell polarization and granzyme production.**  Flow cytometry analysis showing CD16^+^ NK cell frequencies, upon stimulation for 72h with CM derived from (**A**) MIAPaCa2, (n=10; Results are expressed as median and interquartile range, Wilcoxon test, *p < 0.05), (**B**) BxPC3 (n=10; Results are expressed as median and interquartile range, Wilcoxon test, *p < 0.05), (**C**) PANC-1 (n=10; Results are shown as mean ± SD for CD16^+^ NK cells, (**D**) CAFs (n=10; Results are expressed as median and interquartile range, Wilcoxon test, *p < 0.05, **p < 0.01), (**E**) pCAFs (n=5; Results are shown as mean ± SD, Paired t-test, **p < 0.01), treated or not with GAL. Same flow cytometry analysis were also performed to evaluate Granzyme B (GzrB) production by NK cells following 72h stimulation with CM from **(F)** MIAPaCa2 (n=6), **(G)** BxPC3 (n=6), **(H)** PANC-1 (n=6), **(I)** CAFs (n=6), **(J)** pCAFs (n=5), treated or not with GAL. Results are expressed as median and interquartile range, Wilcoxon test, *p < 0.05.

**Supplementary Figure 5**

**Supplementary Figure 5: (A)** *TGFBR1* expression levels in BxPC3, MiaPACA and PANC1 PDAC cell line following 72h silencing using 3 different and specif siRNA sequences (termed seq1, seq2 and seq3). Scramble (Scr) was used as negative control. Results are expressed as 2^-ΔΔCt. 18S was used as reference. Results are shown as mean ± SD. **(B**) *TGFBR1* expression levels in siRNA-treated BxPC3, MiaPACA-2 and PANC1 PDAC cells following a 48-hour washout period. Conditioned media were collected at this time point to prevent carryover of siRNA sequences to the PBMC cultures. Results are expressed as 2^-ΔΔCt. 18S was used as reference. Results are shown as mean ± SD. Flow cytometry analysis showing (**C**) Frequencies of Granzyme B+ (Gzmb+) NK cells, upon stimulation for 72h with CM derived from BxPC3 (green), MiaPACA-2 (orange) and PANC-1 (blue) either silenced (seq1, seq2) or expressing (Scr) TGFBR1. NT (untreated) PBMCs were used as control. For Gzmb+ NK cells treated with BxPC3 CMs, results are expressed as median and interquartile range. Results are expressed median and interquartile range for BxPC3 and as mean ± SD, for MiaPACA-2 and PANC-1.

**Supplementary Figure 6**

**Supplementary Figure 6: (A)** Secretomic array performed on CM of BxPC3, MiaPACA-2 and CAF cell lines treated or not with GAL for 48h **(B)** Membrane array quantified for secretomic analysis (V=vehicle). **(C)** tables showing soluble factors spotted in C7 arrays. (**D**) Phenotypic and functional modulations induced by HGF, IL11, IL17a, VEGFA, and bFGF on NK cells from healthy donors upon 72h stimulation.

**Supplementary Figure 7**

**Supplementary Figure 7: (A)** Box plots showing the expression levels of FGF2, EGFR, HGF, IL11, PLGF and VEGF in PDAC patients (n=179) vs normal tissues (n=171) using data from GEPIA2 (TCGA tumor versus TCGA normal + GTEx normal); Kaplan-Meier curves showing the **(B)** Overall Survival and **(C)** disease free survival in FGF2/EGFR/HGF/IL11/PLGF/VEGF^high^ vs FGF2/EGFR/HGF/IL11/PLGF/VEGF^low^ PDAC patients. **(D)** Correlation analysis showing the positive association between FGF2/EGFR/HGF/IL11/PLGF/VEGF signature (x-axis – 6 signature) and NKP46/CD9 signature (y-axis - 2 signature).

**Supplementary Table 1A: Patient’s clinical features**

| **ID** | **AGE** | **GENDER** | **SURVIVAL*** | **TNM** | **Grading (G1-3)** | **TA** |
| --- | --- | --- | --- | --- | --- | --- |
| PDAC1 | 74 | F | / | pT2N1M0R0 (IIB) | G2 | X |
| PDAC2 | 80 | M | / | NA | NA | X |
| PDAC3 | 80 | F | 11 | pT2N2M0 (III) | G3 | X |
| PDAC4 | 63 | M | 14 | pT2N2M0 (III) | G3 | X |
| PDAC5 | 77 | F | 8 | cT4NxM1 (IV) | NA |  |
| PDAC6 | 57 | M | ? | cT2NxM1 (IV) | NA |  |
| PDAC7 | 78 | M | / | pT2N2M1 (IV) | G3 | X |
| PDAC8 | 80 | M | 3 | pT3N2M1 (IV) | G3 |  |
| PDAC9 | 78 | F | 2,5 | pT2N2M1 (IV) | NA |  |
| PDAC10 | 89 | F | 4 | cT2NxM0 | NA |  |
| PDAC11 | NA | F | 4 | cT4N0 | NA |  |
| PDAC12 | 80 | F | / | pT3N0M0 (IIA) | G3 |  |
| PDAC13 | 74 | F | 2 | cT2N0 | NA |  |
| PDAC14 | 79 | M | / | NA | NA |  |
| PDAC15 | 69 | F | / | NA | NA |  |
| PDAC16 | 69 | M | / | pT2 pN0 | G4 |  |
| PDAC17 | 79 | M | / | pT1c pN1 | G2 |  |
| PDAC18 | 73 | M | / | pT1 pN1 | G3 |  |
| PDAC19 | 71 | F | / | pN1 pN0 M1 | G3 |  |
| PDAC20 | 70 | F | / | pT1a pN0 | G1 |  |
| PDAC21 | 76 | M | / | pT2 pN0 | G2 |  |
| PDAC22 | 53 | F | / | pT2 pN1 | G3 |  |
| PDAC23 | 80 | M | / | pT2 pN0 | G3 |  |
| PDAC24 | 65 | F | / | NA | NA |  |
| PDAC25 | 78 | M | / | NA | NA |  |
| PDAC26 | 78 | F | / | NA | NA |  |

**Age:** 74 ± 8,01 (Mean ± SD), **Male:** 12 (46,15%), **Female:** 14 (53,85%)

NA: Not available

* months from diagnosis

/ still alive

TA: Tissue available

**Supplementary Table 1B: Healthy controls’ clinical features**

| **ID** | **GENDER** | **AGE** |
| --- | --- | --- |
| HC1 | F | 66 |
| HC2 | M | 57 |
| HC3 | F | 64 |
| HC4 | M | 55 |
| HC5 | M | 55 |
| HC6 | M | 57 |
| HC7 | M | 56 |
| HC8 | M | 57 |
| HC9 | M | 54 |
| HC10 | M | 61 |
| HC11 | M | 57 |
| HC12 | F | 57 |
| HC13 | M | 55 |
| HC14 | M | 60 |
| HC15 | M | 57 |
| HC16 | M | 49 |
| HC17 | M | 55 |
| HC18 | M | 54 |
| HC19 | M | 62 |
| HC20 | M | 54 |
| HC21 | M | 59 |
| HC22 | M | 64 |
| HC23 | F | 56 |
| HC24 | F | 55 |
| HC25 | F | 55 |
| **Age:** 57,24 ± 3,81 (Mean ± SD), **Male:** 19/25 (76%), **Female:** 6/25 (24 %) | | |

**Age:** 57,54 ± 4,37 (Mean ± SD), **Male:** 9/13 (69,26 %), **Female:** 4/13 (30,76 %)

**Supplementary Table 2 List of antibodies for flow cytometry**

| **Reactivity** | **Antibody target** | **clone** | **Fluorochrome** | **Supplier** |
| --- | --- | --- | --- | --- |
| human | CD45 | HI30 | BUV395 | BD Bioscience |
| human | CD3 | BW264/56 | PerCp | Miltenyi Biotec |
| human | CD56 | REA196 | APC | Miltenyi Biotec |
| human | CD16 | REA423 | FITC | Miltenyi Biotec |
| human | CD9 | REA1071 | PE | Miltenyi Biotec |
| human | CD49a | 7SR84 | BV421 | BD Bioscience |
| human | CD107a/LAMP-1 | H4A3 | FITC | BD Bioscience |
| human | Perforin | δG9 | PE-CF5694 | BD Bioscience |
| human | Granzyme B | GB11 | PE | BD Bioscience |
| human | IFNγ | 4S.B3 | BV650 | BD Bioscience |
| murine | CD45 | 3O-F11 | BUV395 | BD Bioscience |
| murine | CD3 | 145-2C11 | PerCp | BD Bioscience |
| murine | NK1.1 | PK136 | PE | BD Bioscience |
| murine | CD9 | KMC8 | BUV737 | BD Bioscience |
| murine | CD49a | Ha31/8 | BV421 | BD Bioscience |
| murine | B220 | RA3-6B2 | BV786 | BD Bioscience |
| murine | CD4 | RM4-5 | BV480 | BD Bioscience |
| murine | CD8 | 145-2C11 | BV786 | BD Bioscience |
| murine | IFNγ | XMG1.2 | PECF594 | BD Bioscience |

**Supplementary Table 3: Primer sequences for oligos used for qPCT analysis.**

| **Target** | **Forward sequence** | **Reverse sequence** |
| --- | --- | --- |
| *mVEGF* | CTGCTGTAACGATGAAGCCCTG | GCTGTAGGAAGCTCATCTCTCC |
| *mPlGF* | TGCTGTGGTGATGAAGGTCTGC | GCATTCACAGAGCACATCCTGAG |
| *mSTAT3* | TGTCTACCTCTACCCCGACA | ACATCGGCAGGTCAATGGTA |
| *mCXCR4* | CTATGTGGGCGTCTGGATCC | TCCACAGGCTATCGGGGTAA |
| *mIL-1α* | CAACGTCAAGCAACGGGAAG | AAGGTGCTGATCTGGGTTGG |
| *mIL-1β* | GGCTCATCTGGGATCCTCTC | TCATCTTTTGGGGTCCGTCA |
| *mIL-6* | AGTCCTTCCTACCCCAATTTCC | GGTCTTGGTCCTTAGCCACT |
| *mIL-10* | TGAGGCGCTGTCATCGATTT | GACACCTTGGTCTTGGAGCTT |
| *mIL-17a* | TGACCCCTAAGAAACCCCCA | TCATTGTGGAGGGCAGACAA |
| *mCXCL8* | CCCTGTGACACTCAAGAGCT | CAGTAGCCTTCACCCATGGA |
| *mIFNγ* | CGGCTGACCTAGAGAAGACA | TTTCAATGACTGTGCCGTGG |
| *mTGFβ* | TGATACGCCTGAGTGGCTGTCT | CACAAGAGCAGTGAGCGCTGAA |
| *mGranzyme A* | TTTTGCGAGGCATCACCTCT | TCAGACACAGAAGTGACAGGG |
| *mGranzyme B* | CAGGCCAATGGAACACCTCT | GTGGAGAGGGCAAACTTCCA |
| *mPerforin* | TCTTGGTGGGACTTCAGCTT | CCATACACCTGGCACGAACT |
| *mTigit* | TGCTGTGCTGGGACTCATTT | AGGTTCCATTCCTGTGGCTC |
| *mTIM-3* | CAGGACTCTCCTCTGCCTCT | AACTCTCCACGCTTCTGCTC |
| *mβ-Actin* | TGAGCTGCGTTTTACACCCT | GCCTTCACCGTTCCAGTTTT |
| *mHGF* | GCAGTACCCTCACAAGCATG | ACTCGGATGTTTGGGTCAGT |
| *mVEGFA* | TCGAGGAAAGGGAAAGGGTC | CGCTCCAGGATTTAAACCGG |
| *mVEGFD* | CTTTTGAGCGATCATCCCGG | GAAGCTGCTCGGATCTGTTG |
| *mEGFR* | ACTGACCTCCATGCTTTCGA | CCGCCAAAGAAAACTGACCA |
| *mIL17A* | ACTCTCCACCGCAATGAAGA | CTCTCAGGCTCCCTCTTCAG |
| *mIL11* | TGCTGCTCACACTCACAAAC | CAGGCGACAAACACAGTTCA |
| *mFGF2* | ATGAAGGAAGATGGACGGCT | CAGTTCGTTTCAGTGCCACA |
| *hTGFBR1* | GACAACGTCAGGTTCTGGCTCA | CCGCCACTTTCCTCTCCAAACT |

**Supplementary Table 4: Top 200 differentially expressed genes between TGFBR1^high^VsTGFBR1^low^**

|  | logFC | AveExpr | t | P.Value | adj.P.Val | B |
| --- | --- | --- | --- | --- | --- | --- |
| TGFBR1 | 0,866898 | 6,707114 | 33,03425 | 2,5E-150 | 3,2E-146 | 330,3467 |
| TWSG1 | 0,578819 | 6,499483 | 14,34483 | 1,39E-41 | 8,64E-38 | 83,59492 |
| ITGAV | 0,58341 | 8,608948 | 14,12159 | 1,75E-40 | 7,26E-37 | 81,09464 |
| RAB31 | 0,670631 | 8,332121 | 13,77558 | 8,47E-39 | 2,64E-35 | 77,26538 |
| BMPR2 | 0,464068 | 7,364371 | 13,66709 | 2,83E-38 | 7,05E-35 | 76,07651 |
| CDH11 | 0,926974 | 8,146761 | 13,43841 | 3,52E-37 | 6,55E-34 | 73,58938 |
| PALLD | 0,655165 | 8,699656 | 13,43438 | 3,68E-37 | 6,55E-34 | 73,54585 |
| GPX8 | 0,58934 | 6,679446 | 13,39834 | 5,46E-37 | 8,51E-34 | 73,15625 |
| SEC23A | 0,489253 | 7,199945 | 13,3558 | 8,7E-37 | 1,2E-33 | 72,6973 |
| COL12A1 | 1,044859 | 8,403299 | 13,30036 | 1,59E-36 | 1,99E-33 | 72,10063 |
| RAB23 | 0,688402 | 5,433078 | 13,16565 | 6,88E-36 | 7,8E-33 | 70,6571 |
| CSGALNACT2 | 0,458079 | 6,570732 | 13,14735 | 8,39E-36 | 8,72E-33 | 70,46174 |
| ANTXR1 | 0,753823 | 7,531433 | 13,10919 | 1,27E-35 | 1,22E-32 | 70,05486 |
| GNB4 | 0,54837 | 6,233873 | 12,99993 | 4,11E-35 | 3,66E-32 | 68,89414 |
| SPIN1 | 0,310449 | 7,311005 | 12,80391 | 3,35E-34 | 2,78E-31 | 66,82728 |
| COL5A2 | 0,843827 | 8,441791 | 12,77148 | 4,73E-34 | 3,52E-31 | 66,48726 |
| SLC44A1 | 0,447028 | 7,89814 | 12,77007 | 4,8E-34 | 3,52E-31 | 66,4725 |
| CTHRC1 | 0,911022 | 8,013588 | 12,69184 | 1,1E-33 | 7,61E-31 | 65,65471 |
| SGCB | 0,453293 | 6,95336 | 12,63801 | 1,94E-33 | 1,27E-30 | 65,09403 |
| THBS2 | 1,009456 | 8,677321 | 12,45535 | 1,32E-32 | 8,24E-30 | 63,20282 |
| CALU | 0,49794 | 8,004674 | 12,40796 | 2,17E-32 | 1,23E-29 | 62,71513 |
| OSBPL8 | 0,394954 | 6,597173 | 12,40788 | 2,17E-32 | 1,23E-29 | 62,71429 |
| VCL | 0,411372 | 7,690799 | 12,38327 | 2,81E-32 | 1,52E-29 | 62,46154 |
| SOCS5 | 0,369928 | 6,020728 | 12,3457 | 4,15E-32 | 2,15E-29 | 62,07628 |
| UBE2Q2 | 0,37731 | 6,181611 | 12,32818 | 4,98E-32 | 2,48E-29 | 61,89698 |
| TM7SF2 | -0,41359 | 5,7725 | -12,2739 | 8,73E-32 | 4,19E-29 | 61,34251 |
| EDIL3 | 0,873185 | 6,837032 | 12,25383 | 1,08E-31 | 4,96E-29 | 61,13764 |
| EDNRA | 0,699563 | 6,80564 | 12,16456 | 2,7E-31 | 1,2E-28 | 60,23011 |
| CLIC4 | 0,52528 | 8,074334 | 12,14372 | 3,35E-31 | 1,44E-28 | 60,01885 |
| MSRB3 | 0,635418 | 6,234225 | 12,12876 | 3,9E-31 | 1,62E-28 | 59,86733 |
| KPNA3 | 0,329575 | 6,250797 | 12,10604 | 4,93E-31 | 1,98E-28 | 59,63762 |
| DDR2 | 0,63906 | 6,405159 | 12,10229 | 5,12E-31 | 1,99E-28 | 59,59962 |
| LTBP1 | 0,635305 | 7,693068 | 12,09576 | 5,47E-31 | 2,07E-28 | 59,53371 |
| GNAQ | 0,308957 | 6,98981 | 12,09048 | 5,78E-31 | 2,12E-28 | 59,48036 |
| KCTD10 | 0,378281 | 7,138496 | 12,07918 | 6,49E-31 | 2,31E-28 | 59,36625 |
| ENTPD1 | 0,545373 | 6,652658 | 12,06869 | 7,22E-31 | 2,5E-28 | 59,26038 |
| FNDC1 | 1,093476 | 7,481561 | 12,03609 | 1,01E-30 | 3,4E-28 | 58,93185 |
| LUM | 0,843453 | 10,0283 | 12,03028 | 1,07E-30 | 3,51E-28 | 58,87336 |
| PPIC | 0,403875 | 7,786345 | 12,0108 | 1,31E-30 | 4,17E-28 | 58,67744 |
| SPARC | 0,782242 | 11,00413 | 11,99286 | 1,57E-30 | 4,88E-28 | 58,49716 |
| POSTN | 1,142244 | 8,883529 | 11,95581 | 2,29E-30 | 6,95E-28 | 58,12547 |
| SLC30A7 | 0,285583 | 6,630681 | 11,93511 | 2,82E-30 | 8,37E-28 | 57,91806 |
| SKIL | 0,447689 | 7,024236 | 11,91425 | 3,49E-30 | 1,01E-27 | 57,70936 |
| STK38L | 0,446036 | 7,155575 | 11,90201 | 3,95E-30 | 1,12E-27 | 57,58708 |
| FN1 | 0,943838 | 10,1359 | 11,85717 | 6,22E-30 | 1,72E-27 | 57,13968 |
| ATXN1 | 0,357904 | 6,681423 | 11,77999 | 1,35E-29 | 3,61E-27 | 56,37228 |
| DEGS1 | 0,370308 | 6,801011 | 11,77944 | 1,36E-29 | 3,61E-27 | 56,36678 |
| COL3A1 | 0,831993 | 11,0947 | 11,75632 | 1,72E-29 | 4,46E-27 | 56,13759 |
| UBTD2 | 0,281094 | 5,97311 | 11,65697 | 4,66E-29 | 1,18E-26 | 55,15628 |
| MXRA5 | 0,72518 | 8,929323 | 11,6332 | 5,9E-29 | 1,47E-26 | 54,92236 |
| GPR137B | 0,416614 | 6,837628 | 11,60976 | 7,46E-29 | 1,82E-26 | 54,69208 |
| PARVA | 0,356475 | 7,89535 | 11,55227 | 1,32E-28 | 3,17E-26 | 54,12853 |
| ASPN | 0,903266 | 8,199672 | 11,52659 | 1,71E-28 | 4,01E-26 | 53,8774 |
| FBN1 | 0,80061 | 8,255082 | 11,39383 | 6,33E-28 | 1,46E-25 | 52,5856 |
| CTTNBP2NL | 0,312106 | 6,139843 | 11,39163 | 6,47E-28 | 1,47E-25 | 52,56423 |
| ANKH | 0,415103 | 6,923531 | 11,3613 | 8,72E-28 | 1,94E-25 | 52,27071 |
| ZNF444 | -0,36573 | 6,865788 | -11,3371 | 1,11E-27 | 2,42E-25 | 52,03663 |
| RAP2A | 0,373659 | 6,252363 | 11,33214 | 1,16E-27 | 2,49E-25 | 51,98891 |
| TMTC3 | 0,345508 | 5,9422 | 11,27968 | 1,94E-27 | 4,1E-25 | 51,48335 |
| CAP1 | 0,393495 | 8,607669 | 11,27566 | 2,02E-27 | 4,18E-25 | 51,44473 |
| GREM1 | 1,104122 | 8,306215 | 11,27432 | 2,04E-27 | 4,18E-25 | 51,43179 |
| PRSS23 | 0,55167 | 7,690401 | 11,24475 | 2,73E-27 | 5,45E-25 | 51,14774 |
| PLS3 | 0,452261 | 8,018488 | 11,24381 | 2,75E-27 | 5,45E-25 | 51,13865 |
| SPOCK1 | 0,73756 | 6,140591 | 11,22229 | 3,39E-27 | 6,61E-25 | 50,93227 |
| VCAN | 0,764894 | 9,4434 | 11,21427 | 3,67E-27 | 6,95E-25 | 50,85536 |
| CTSK | 0,768472 | 8,864678 | 11,21387 | 3,68E-27 | 6,95E-25 | 50,85156 |
| ADAMTS12 | 0,605836 | 5,963723 | 11,2124 | 3,74E-27 | 6,95E-25 | 50,83748 |
| DPYSL3 | 0,644558 | 8,008495 | 11,16693 | 5,81E-27 | 1,07E-24 | 50,40249 |
| CORO1C | 0,371093 | 7,660376 | 11,14976 | 6,87E-27 | 1,23E-24 | 50,23858 |
| FAM114A1 | 0,337451 | 6,870816 | 11,1495 | 6,88E-27 | 1,23E-24 | 50,2361 |
| PLXDC2 | 0,613039 | 8,214512 | 11,12498 | 8,73E-27 | 1,53E-24 | 50,00236 |
| ARF4 | 0,371892 | 8,831351 | 11,12103 | 9,07E-27 | 1,57E-24 | 49,96479 |
| MFAP3 | 0,362241 | 5,744646 | 11,10362 | 1,07E-26 | 1,83E-24 | 49,79903 |
| ACTR3 | 0,313682 | 8,799623 | 11,05048 | 1,79E-26 | 3,02E-24 | 49,29443 |
| YIPF5 | 0,349474 | 6,826019 | 11,03977 | 1,99E-26 | 3,3E-24 | 49,19288 |
| ACTR2 | 0,34212 | 8,23445 | 10,99786 | 2,97E-26 | 4,87E-24 | 48,79642 |
| PRKD1 | 0,515122 | 5,544292 | 10,97284 | 3,78E-26 | 6,12E-24 | 48,56035 |
| EFR3A | 0,321313 | 7,725397 | 10,9488 | 4,76E-26 | 7,6E-24 | 48,33376 |
| PRKG1 | 0,447223 | 5,174706 | 10,94705 | 4,84E-26 | 7,63E-24 | 48,31732 |
| LPP | 0,418408 | 7,881743 | 10,9422 | 5,07E-26 | 7,89E-24 | 48,27164 |
| SPRED1 | 0,341628 | 6,560262 | 10,91536 | 6,55E-26 | 1,01E-23 | 48,0193 |
| SFT2D2 | 0,352944 | 6,608392 | 10,89233 | 8,15E-26 | 1,24E-23 | 47,8031 |
| JAM3 | 0,505957 | 6,213385 | 10,8762 | 9,51E-26 | 1,43E-23 | 47,65182 |
| PICALM | 0,288214 | 7,963127 | 10,8601 | 1,11E-25 | 1,64E-23 | 47,50105 |
| MTMR2 | 0,277031 | 6,746464 | 10,85142 | 1,2E-25 | 1,77E-23 | 47,41987 |
| ATP2A2 | 0,309061 | 7,807602 | 10,83852 | 1,36E-25 | 1,97E-23 | 47,29919 |
| SGCD | 0,598337 | 5,077589 | 10,81301 | 1,73E-25 | 2,48E-23 | 47,06103 |
| CPSF4 | -0,27627 | 6,343802 | -10,7775 | 2,43E-25 | 3,44E-23 | 46,73033 |
| FSTL1 | 0,599192 | 9,280271 | 10,7568 | 2,95E-25 | 4,13E-23 | 46,53754 |
| ENTPD7 | 0,336547 | 5,703928 | 10,75299 | 3,06E-25 | 4,24E-23 | 46,50221 |
| TNFAIP6 | 0,758172 | 5,884283 | 10,75183 | 3,09E-25 | 4,24E-23 | 46,49138 |
| ZEB1 | 0,488072 | 6,530194 | 10,74079 | 3,43E-25 | 4,65E-23 | 46,38886 |
| MACROD1 | -0,37703 | 5,663646 | -10,7224 | 4,08E-25 | 5,47E-23 | 46,2178 |
| ZMAT3 | 0,372645 | 6,221644 | 10,71429 | 4,41E-25 | 5,84E-23 | 46,14304 |
| RAI14 | 0,445616 | 6,791047 | 10,69963 | 5,06E-25 | 6,64E-23 | 46,00725 |
| FAP | 0,809703 | 7,941506 | 10,68591 | 5,75E-25 | 7,47E-23 | 45,88032 |
| HTRA1 | 0,609997 | 8,998871 | 10,68344 | 5,89E-25 | 7,57E-23 | 45,85744 |
| AGPS | 0,274067 | 6,415587 | 10,67767 | 6,22E-25 | 7,91E-23 | 45,8041 |
| IL13RA1 | 0,264269 | 7,90009 | 10,65504 | 7,69E-25 | 9,68E-23 | 45,59511 |
| RHOA | 0,306235 | 9,577883 | 10,65341 | 7,81E-25 | 9,73E-23 | 45,58011 |
| PLXNC1 | 0,462247 | 5,763872 | 10,62758 | 9,95E-25 | 1,23E-22 | 45,3419 |
| PJA2 | 0,339625 | 7,451715 | 10,60524 | 1,23E-24 | 1,5E-22 | 45,13637 |
| EXOC5 | 0,277625 | 5,342999 | 10,60294 | 1,25E-24 | 1,52E-22 | 45,11517 |
| BRF1 | -0,22337 | 5,832049 | -10,5975 | 1,32E-24 | 1,58E-22 | 45,06502 |
| FBXO32 | 0,626533 | 7,518801 | 10,59447 | 1,36E-24 | 1,61E-22 | 45,03727 |
| OSTM1 | 0,322602 | 5,792952 | 10,58831 | 1,44E-24 | 1,69E-22 | 44,9807 |
| PDLIM3 | 0,653775 | 7,040154 | 10,57083 | 1,69E-24 | 1,97E-22 | 44,82023 |
| COMTD1 | -0,49287 | 6,773267 | -10,5488 | 2,08E-24 | 2,4E-22 | 44,61828 |
| GNPTAB | 0,27737 | 6,31378 | 10,54623 | 2,13E-24 | 2,43E-22 | 44,59478 |
| DACT1 | 0,474321 | 6,500786 | 10,54516 | 2,15E-24 | 2,43E-22 | 44,58497 |
| COL4A1 | 0,52655 | 8,398076 | 10,52025 | 2,71E-24 | 3,04E-22 | 44,357 |
| TPM4 | 0,334014 | 9,06909 | 10,50432 | 3,14E-24 | 3,49E-22 | 44,21152 |
| SH3PXD2B | 0,412343 | 7,059757 | 10,49928 | 3,29E-24 | 3,63E-22 | 44,1655 |
| SKAP2 | 0,395705 | 7,274156 | 10,47993 | 3,93E-24 | 4,3E-22 | 43,98894 |
| GNG12 | 0,341453 | 7,590622 | 10,44109 | 5,64E-24 | 6,11E-22 | 43,63546 |
| LOX | 0,673848 | 6,274827 | 10,41778 | 6,99E-24 | 7,51E-22 | 43,42367 |
| PCP2 | -0,34509 | 4,31672 | -10,41 | 7,51E-24 | 7,94E-22 | 43,35296 |
| RAB1A | 0,304219 | 8,225176 | 10,40995 | 7,51E-24 | 7,94E-22 | 43,35272 |
| RASSF8 | 0,392407 | 5,58795 | 10,40519 | 7,85E-24 | 8,23E-22 | 43,30949 |
| SETD7 | 0,321847 | 6,92086 | 10,38819 | 9,18E-24 | 9,54E-22 | 43,15546 |
| MORF4L1 | 0,304542 | 8,343722 | 10,38624 | 9,35E-24 | 9,63E-22 | 43,13783 |
| PXDN | 0,631385 | 7,223726 | 10,38502 | 9,45E-24 | 9,66E-22 | 43,12678 |
| NOX4 | 0,710539 | 5,647108 | 10,36972 | 1,09E-23 | 1,1E-21 | 42,98831 |
| FRMD6 | 0,524491 | 6,24654 | 10,34474 | 1,37E-23 | 1,38E-21 | 42,76267 |
| APBB2 | 0,352922 | 6,814041 | 10,33599 | 1,48E-23 | 1,48E-21 | 42,68374 |
| COL5A1 | 0,658659 | 8,625129 | 10,3248 | 1,64E-23 | 1,62E-21 | 42,58277 |
| LAMC1 | 0,40242 | 8,100247 | 10,32469 | 1,65E-23 | 1,62E-21 | 42,5818 |
| COL6A3 | 0,578418 | 9,289901 | 10,29146 | 2,23E-23 | 2,17E-21 | 42,28265 |
| AGER | -0,3881 | 5,217038 | -10,2904 | 2,25E-23 | 2,18E-21 | 42,27275 |
| CEP170 | 0,377154 | 5,695226 | 10,27866 | 2,51E-23 | 2,4E-21 | 42,1676 |
| RAB8B | 0,333929 | 6,592723 | 10,27412 | 2,61E-23 | 2,49E-21 | 42,12686 |
| MICAL2 | 0,425238 | 7,11428 | 10,21112 | 4,64E-23 | 4,38E-21 | 41,56238 |
| THBS1 | 0,717074 | 8,958773 | 10,20294 | 5E-23 | 4,69E-21 | 41,4893 |
| ZBTB38 | 0,31356 | 7,349253 | 10,20126 | 5,08E-23 | 4,72E-21 | 41,47431 |
| BCAT2 | -0,2682 | 6,00438 | -10,1983 | 5,21E-23 | 4,81E-21 | 41,44827 |
| PRRC1 | 0,274313 | 7,185692 | 10,19755 | 5,25E-23 | 4,81E-21 | 41,44116 |
| BAG2 | 0,570574 | 5,319337 | 10,1884 | 5,7E-23 | 5,19E-21 | 41,35956 |
| ZMPSTE24 | 0,29298 | 7,593494 | 10,18177 | 6,06E-23 | 5,47E-21 | 41,30037 |
| KCTD20 | 0,315866 | 6,65729 | 10,18039 | 6,13E-23 | 5,5E-21 | 41,28809 |
| LACTB | 0,339148 | 5,929017 | 10,17486 | 6,45E-23 | 5,74E-21 | 41,23878 |
| TMEM167A | 0,307788 | 7,007625 | 10,16729 | 6,91E-23 | 6,11E-21 | 41,17128 |
| GLIPR1 | 0,485806 | 6,614089 | 10,16401 | 7,12E-23 | 6,25E-21 | 41,14213 |
| FEM1C | 0,30768 | 5,935031 | 10,15867 | 7,47E-23 | 6,51E-21 | 41,09454 |
| BNC2 | 0,55727 | 5,435799 | 10,15452 | 7,75E-23 | 6,71E-21 | 41,05765 |
| ATP6AP2 | 0,34886 | 7,726394 | 10,15374 | 7,81E-23 | 6,71E-21 | 41,0507 |
| TMEM145 | -0,40339 | 4,054507 | -10,144 | 8,53E-23 | 7,24E-21 | 40,96439 |
| VAMP7 | 0,292925 | 6,862335 | 10,14388 | 8,54E-23 | 7,24E-21 | 40,96296 |
| LAPTM4A | 0,309876 | 9,756368 | 10,12613 | 1E-22 | 8,44E-21 | 40,80522 |
| PIK3CA | 0,293741 | 6,136004 | 10,1108 | 1,15E-22 | 9,61E-21 | 40,66912 |
| HEXIM2 | -0,28341 | 5,34683 | -10,1103 | 1,16E-22 | 9,61E-21 | 40,66459 |
| TMUB1 | -0,33183 | 6,8005 | -10,1087 | 1,17E-22 | 9,69E-21 | 40,65056 |
| MAP4K5 | 0,276627 | 6,700105 | 10,1012 | 1,26E-22 | 1,03E-20 | 40,58401 |
| LIN7B | -0,32882 | 4,961892 | -10,079 | 1,53E-22 | 1,25E-20 | 40,38698 |
| PDLIM5 | 0,309365 | 7,718907 | 10,07387 | 1,61E-22 | 1,3E-20 | 40,34201 |
| TSHZ3 | 0,508508 | 6,256338 | 10,07081 | 1,65E-22 | 1,33E-20 | 40,3149 |
| DNAJC10 | 0,275544 | 7,661374 | 10,06921 | 1,67E-22 | 1,34E-20 | 40,30078 |
| FERMT2 | 0,544477 | 6,688544 | 10,06196 | 1,79E-22 | 1,42E-20 | 40,2367 |
| COL11A1 | 1,220799 | 7,326587 | 10,06098 | 1,8E-22 | 1,42E-20 | 40,22803 |
| ACAP2 | 0,269132 | 6,640676 | 10,05471 | 1,91E-22 | 1,5E-20 | 40,17261 |
| BTBD1 | 0,277899 | 7,419258 | 10,04687 | 2,05E-22 | 1,59E-20 | 40,10343 |
| PRODH2 | -0,39184 | 3,378106 | -10,0374 | 2,23E-22 | 1,73E-20 | 40,01949 |
| VAMP3 | 0,275639 | 7,393047 | 10,03357 | 2,31E-22 | 1,77E-20 | 39,98608 |
| NEDD4 | 0,331979 | 5,226073 | 10,02924 | 2,4E-22 | 1,83E-20 | 39,94785 |
| ENAH | 0,367592 | 6,739481 | 10,02127 | 2,58E-22 | 1,96E-20 | 39,87761 |
| FKBP14 | 0,375771 | 5,750597 | 10,0177 | 2,66E-22 | 2,01E-20 | 39,84615 |
| COPS8 | 0,285065 | 6,373722 | 10,01408 | 2,75E-22 | 2,06E-20 | 39,81429 |
| RAP1A | 0,344506 | 6,979508 | 9,991473 | 3,36E-22 | 2,51E-20 | 39,61541 |
| SLAIN2 | 0,217322 | 6,657792 | 9,977526 | 3,81E-22 | 2,83E-20 | 39,49288 |
| TRAM2 | 0,330665 | 6,652802 | 9,971079 | 4,04E-22 | 2,98E-20 | 39,43628 |
| PDCL | 0,232632 | 5,864308 | 9,965965 | 4,22E-22 | 3,1E-20 | 39,39141 |
| ANKRD23 | -0,31346 | 4,387317 | -9,96167 | 4,39E-22 | 3,2E-20 | 39,3537 |
| SEC24A | 0,288481 | 6,145014 | 9,949817 | 4,88E-22 | 3,54E-20 | 39,24983 |
| PRKAR1A | 0,304427 | 8,027884 | 9,945918 | 5,05E-22 | 3,64E-20 | 39,21567 |
| ACOT9 | 0,242825 | 7,078509 | 9,944314 | 5,12E-22 | 3,67E-20 | 39,20163 |
| TMEM30A | 0,332889 | 7,499225 | 9,92464 | 6,1E-22 | 4,35E-20 | 39,02944 |
| PAFAH1B1 | 0,200963 | 7,437567 | 9,918024 | 6,47E-22 | 4,59E-20 | 38,9716 |
| PHLDB3 | -0,31517 | 5,59069 | -9,91027 | 6,94E-22 | 4,89E-20 | 38,90382 |
| LOXL2 | 0,656075 | 6,906835 | 9,906656 | 7,16E-22 | 5,02E-20 | 38,87229 |
| MFSD1 | 0,344317 | 7,963191 | 9,899664 | 7,62E-22 | 5,31E-20 | 38,81125 |
| TLR4 | 0,447753 | 5,862938 | 9,897158 | 7,79E-22 | 5,4E-20 | 38,78938 |
| PTGFRN | 0,340088 | 7,019871 | 9,895253 | 7,93E-22 | 5,46E-20 | 38,77276 |
| DDX51 | -0,26012 | 5,700693 | -9,8685 | 1E-21 | 6,88E-20 | 38,53962 |
| WDR47 | 0,289913 | 5,01562 | 9,860892 | 1,08E-21 | 7,32E-20 | 38,47337 |
| TMEM165 | 0,275965 | 7,418935 | 9,850167 | 1,18E-21 | 8,01E-20 | 38,38009 |
| KLHL5 | 0,381974 | 6,055413 | 9,838928 | 1,31E-21 | 8,79E-20 | 38,28242 |
| PEA15 | 0,306693 | 8,310451 | 9,838418 | 1,31E-21 | 8,79E-20 | 38,27799 |
| NID1 | 0,47775 | 6,841878 | 9,824392 | 1,48E-21 | 9,9E-20 | 38,15623 |
| RECQL | 0,328287 | 5,768212 | 9,818435 | 1,56E-21 | 1,04E-19 | 38,10456 |
| NIPSNAP3A | 0,285993 | 6,152749 | 9,815439 | 1,61E-21 | 1,06E-19 | 38,07858 |
| MYO1E | 0,352379 | 7,529758 | 9,813512 | 1,63E-21 | 1,07E-19 | 38,06188 |
| DKK3 | 0,463477 | 7,041969 | 9,80547 | 1,75E-21 | 1,14E-19 | 37,99219 |
| CYP1A2 | -0,28845 | 3,633346 | -9,80175 | 1,81E-21 | 1,17E-19 | 37,95999 |
| CTSB | 0,388013 | 9,885436 | 9,801725 | 1,81E-21 | 1,17E-19 | 37,95975 |
| MYLK | 0,666066 | 7,840127 | 9,800165 | 1,84E-21 | 1,18E-19 | 37,94624 |
| MMP2 | 0,66543 | 8,29176 | 9,798185 | 1,87E-21 | 1,19E-19 | 37,92909 |
| PIF1 | -0,31569 | 4,547024 | -9,79818 | 1,87E-21 | 1,19E-19 | 37,92902 |
| PSD3 | 0,384047 | 6,300557 | 9,795269 | 1,92E-21 | 1,21E-19 | 37,90385 |
| MAP3K2 | 0,216231 | 6,80534 | 9,794453 | 1,93E-21 | 1,22E-19 | 37,89678 |
| RAP1B | 0,311719 | 7,817693 | 9,793137 | 1,96E-21 | 1,23E-19 | 37,88539 |
| MTMR6 | 0,252703 | 5,964449 | 9,781517 | 2,17E-21 | 1,35E-19 | 37,78487 |

**Supplementary Table 5: Top 200 differentially expressed genes between TGFB1^high^VsTGFB1^low^**

|  | logFC | AveExpr | t | P.Value | adj.P.Val | B |
| --- | --- | --- | --- | --- | --- | --- |
| TGFB1 | 0,836614 | 6,648634 | 35,97389 | 9,2E-168 | 1,1E-163 | 370,5527 |
| GNAI2 | 0,474484 | 7,790652 | 17,79633 | 1,03E-59 | 6,43E-56 | 124,9223 |
| BGN | 0,765194 | 9,329775 | 16,23584 | 2,8E-51 | 1,16E-47 | 105,7135 |
| ZYX | 0,498007 | 7,800126 | 16,1334 | 9,73E-51 | 3,03E-47 | 104,4816 |
| CMTM3 | 0,663666 | 7,38281 | 16,00047 | 4,87E-50 | 1,21E-46 | 102,8888 |
| LGALS1 | 0,757577 | 9,419154 | 15,64843 | 3,36E-48 | 6,97E-45 | 98,70283 |
| TGFB1I1 | 0,609235 | 6,598273 | 15,50453 | 1,87E-47 | 3,32E-44 | 97,0057 |
| COL6A2 | 0,811543 | 8,313474 | 15,18538 | 8,15E-46 | 1,27E-42 | 93,27121 |
| RCN3 | 0,663604 | 6,871684 | 15,09672 | 2,31E-45 | 3,2E-42 | 92,24113 |
| ARL4C | 0,734079 | 7,407533 | 15,06354 | 3,41E-45 | 4,25E-42 | 91,85649 |
| CHST11 | 0,660294 | 6,847862 | 14,92152 | 1,79E-44 | 2,03E-41 | 90,21534 |
| MFGE8 | 0,527051 | 7,097009 | 14,84113 | 4,57E-44 | 4,75E-41 | 89,29021 |
| AXL | 0,690033 | 7,205335 | 14,73711 | 1,53E-43 | 1,46E-40 | 88,09721 |
| COL5A1 | 0,885384 | 8,625129 | 14,715 | 1,97E-43 | 1,76E-40 | 87,84424 |
| LOXL1 | 0,74785 | 7,552125 | 14,6316 | 5,17E-43 | 4,05E-40 | 86,89204 |
| MYH9 | 0,483876 | 9,622713 | 14,63119 | 5,19E-43 | 4,05E-40 | 86,88729 |
| FN1 | 1,107643 | 10,1359 | 14,41447 | 6,25E-42 | 4,59E-39 | 84,42729 |
| SERPINH1 | 0,620333 | 8,110074 | 14,13093 | 1,57E-40 | 1,09E-37 | 81,24066 |
| AEBP1 | 0,858437 | 9,362408 | 14,10312 | 2,15E-40 | 1,41E-37 | 80,93012 |
| FLNA | 0,757272 | 8,848213 | 14,09461 | 2,37E-40 | 1,48E-37 | 80,8352 |
| SPARC | 0,888966 | 11,00413 | 14,01145 | 6,04E-40 | 3,59E-37 | 79,9091 |
| AP1M1 | 0,314036 | 6,496276 | 13,98253 | 8,37E-40 | 4,74E-37 | 79,58778 |
| EFEMP2 | 0,569506 | 7,415895 | 13,93741 | 1,39E-39 | 7,52E-37 | 79,08728 |
| CTSB | 0,520695 | 9,885436 | 13,85521 | 3,48E-39 | 1,81E-36 | 78,17797 |
| COL6A1 | 0,678755 | 8,500456 | 13,8507 | 3,66E-39 | 1,83E-36 | 78,12816 |
| COL5A2 | 0,891837 | 8,441791 | 13,66681 | 2,84E-38 | 1,36E-35 | 76,10604 |
| PLAU | 0,998523 | 8,384408 | 13,60581 | 5,57E-38 | 2,57E-35 | 75,43897 |
| AP2M1 | 0,388394 | 7,93675 | 13,53008 | 1,28E-37 | 5,72E-35 | 74,61327 |
| MRC2 | 0,565184 | 6,81735 | 13,50641 | 1,67E-37 | 7,17E-35 | 74,35578 |
| PDLIM7 | 0,537 | 7,137685 | 13,50184 | 1,75E-37 | 7,28E-35 | 74,30611 |
| GNA12 | 0,308264 | 6,80503 | 13,29924 | 1,61E-36 | 6,48E-34 | 72,11404 |
| COL1A1 | 0,90765 | 11,62191 | 13,24213 | 3E-36 | 1,17E-33 | 71,49992 |
| RAB31 | 0,649308 | 8,332121 | 13,23727 | 3,16E-36 | 1,2E-33 | 71,44778 |
| MAP7D1 | 0,354165 | 7,73213 | 13,20059 | 4,71E-36 | 1,73E-33 | 71,0543 |
| MYO9B | 0,334384 | 7,022545 | 13,16145 | 7,2E-36 | 2,57E-33 | 70,63514 |
| COL3A1 | 0,913322 | 11,0947 | 13,1471 | 8,41E-36 | 2,91E-33 | 70,48168 |
| PXDN | 0,770188 | 7,223726 | 13,11904 | 1,14E-35 | 3,84E-33 | 70,18189 |
| COL4A2 | 0,659175 | 9,542295 | 13,05633 | 2,24E-35 | 7,35E-33 | 69,51343 |
| BMP1 | 0,416685 | 6,01144 | 13,04798 | 2,45E-35 | 7,84E-33 | 69,42458 |
| FAP | 0,958042 | 7,941506 | 13,03152 | 2,93E-35 | 9,13E-33 | 69,24953 |
| LOX | 0,814378 | 6,274827 | 13,015 | 3,5E-35 | 1,06E-32 | 69,07404 |
| NRBP1 | 0,272536 | 7,322859 | 12,92887 | 8,81E-35 | 2,62E-32 | 68,16105 |
| VIM | 0,590158 | 10,13449 | 12,82861 | 2,57E-34 | 7,46E-32 | 67,10337 |
| CTHRC1 | 0,918155 | 8,013588 | 12,81232 | 3,06E-34 | 8,67E-32 | 66,93197 |
| VCAN | 0,855754 | 9,4434 | 12,80949 | 3,15E-34 | 8,73E-32 | 66,90224 |
| THY1 | 0,694574 | 8,743692 | 12,77729 | 4,44E-34 | 1,2E-31 | 66,56399 |
| OLFML2B | 0,725624 | 7,412157 | 12,76936 | 4,83E-34 | 1,28E-31 | 66,48071 |
| PLXND1 | 0,426494 | 7,094414 | 12,74759 | 6,09E-34 | 1,58E-31 | 66,25246 |
| PI4K2A | 0,297642 | 6,151037 | 12,71588 | 8,52E-34 | 2,17E-31 | 65,92034 |
| SULF2 | 0,652972 | 8,818812 | 12,69106 | 1,11E-33 | 2,76E-31 | 65,66086 |
| ARHGAP1 | 0,337599 | 7,955859 | 12,68233 | 1,22E-33 | 2,97E-31 | 65,5696 |
| COL4A1 | 0,616205 | 8,398076 | 12,64805 | 1,75E-33 | 4,19E-31 | 65,21186 |
| CTSZ | 0,554783 | 8,490733 | 12,6265 | 2,19E-33 | 5,02E-31 | 64,98732 |
| LRP1 | 0,490112 | 8,184266 | 12,62581 | 2,21E-33 | 5,02E-31 | 64,98012 |
| LOXL2 | 0,808374 | 6,906835 | 12,62552 | 2,21E-33 | 5,02E-31 | 64,97711 |
| MED15 | 0,334307 | 7,301481 | 12,61049 | 2,59E-33 | 5,78E-31 | 64,82068 |
| SLC39A13 | 0,3763 | 7,765122 | 12,60651 | 2,71E-33 | 5,92E-31 | 64,77926 |
| ASAP1 | 0,405423 | 6,567296 | 12,59646 | 3,01E-33 | 6,47E-31 | 64,67469 |
| SRPX2 | 0,712836 | 7,438043 | 12,58876 | 3,26E-33 | 6,89E-31 | 64,59462 |
| CNN2 | 0,437574 | 8,284229 | 12,58175 | 3,51E-33 | 7,3E-31 | 64,52177 |
| TIMP1 | 0,535777 | 10,18257 | 12,57372 | 3,82E-33 | 7,81E-31 | 64,43838 |
| ADAM19 | 0,592546 | 6,41302 | 12,56523 | 4,18E-33 | 8,4E-31 | 64,35025 |
| CD248 | 0,693437 | 7,520608 | 12,56052 | 4,39E-33 | 8,69E-31 | 64,30136 |
| EMP3 | 0,54938 | 7,098003 | 12,55444 | 4,68E-33 | 9,12E-31 | 64,23826 |
| MXRA8 | 0,61166 | 8,69189 | 12,54278 | 5,29E-33 | 1,01E-30 | 64,11729 |
| MMP2 | 0,823228 | 8,29176 | 12,53861 | 5,53E-33 | 1,04E-30 | 64,07409 |
| RAC2 | 0,534339 | 7,15271 | 12,52737 | 6,22E-33 | 1,16E-30 | 63,95754 |
| BICD2 | 0,331957 | 5,971422 | 12,49942 | 8,34E-33 | 1,53E-30 | 63,66815 |
| ANGPTL2 | 0,67885 | 6,961484 | 12,46763 | 1,16E-32 | 2,1E-30 | 63,33955 |
| POSTN | 1,182613 | 8,883529 | 12,46115 | 1,24E-32 | 2,22E-30 | 63,27264 |
| SPOCK1 | 0,805174 | 6,140591 | 12,44567 | 1,46E-32 | 2,57E-30 | 63,11285 |
| SPON2 | 0,707964 | 8,590181 | 12,41785 | 1,96E-32 | 3,37E-30 | 62,82615 |
| CD276 | 0,37503 | 6,968679 | 12,4171 | 1,97E-32 | 3,37E-30 | 62,81844 |
| EMILIN1 | 0,633656 | 7,669436 | 12,40656 | 2,2E-32 | 3,71E-30 | 62,70981 |
| TOM1 | 0,330856 | 6,608264 | 12,38274 | 2,82E-32 | 4,69E-30 | 62,46479 |
| WDR1 | 0,332345 | 8,753848 | 12,36957 | 3,23E-32 | 5,31E-30 | 62,32947 |
| FKBP10 | 0,564741 | 7,793443 | 12,36342 | 3,45E-32 | 5,58E-30 | 62,26632 |
| ITGB5 | 0,560105 | 8,452968 | 12,32925 | 4,92E-32 | 7,86E-30 | 61,91566 |
| SERPINE1 | 0,884345 | 8,296905 | 12,30057 | 6,62E-32 | 1,05E-29 | 61,62183 |
| P4HA3 | 0,685639 | 5,328385 | 12,29496 | 7,02E-32 | 1,09E-29 | 61,56445 |
| COL6A3 | 0,67383 | 9,289901 | 12,29127 | 7,29E-32 | 1,12E-29 | 61,5267 |
| CDH11 | 0,860773 | 8,146761 | 12,28443 | 7,83E-32 | 1,19E-29 | 61,45671 |
| PTPN1 | 0,356189 | 7,151348 | 12,28285 | 7,96E-32 | 1,2E-29 | 61,44058 |
| LAPTM5 | 0,659184 | 8,171773 | 12,24284 | 1,2E-31 | 1,78E-29 | 61,03194 |
| FSTL1 | 0,66927 | 9,280271 | 12,24218 | 1,21E-31 | 1,78E-29 | 61,02518 |
| MSN | 0,470555 | 8,39098 | 12,21636 | 1,58E-31 | 2,29E-29 | 60,76196 |
| TPP1 | 0,354474 | 7,443034 | 12,17786 | 2,35E-31 | 3,37E-29 | 60,37014 |
| FSCN1 | 0,539338 | 6,622653 | 12,17494 | 2,43E-31 | 3,44E-29 | 60,34038 |
| ARPC1B | 0,464749 | 7,270194 | 12,15798 | 2,89E-31 | 4,05E-29 | 60,16807 |
| GFPT2 | 0,764643 | 7,066876 | 12,1421 | 3,4E-31 | 4,71E-29 | 60,00687 |
| LEF1 | 0,606129 | 5,924937 | 12,13391 | 3,7E-31 | 5,07E-29 | 59,92385 |
| RHOC | 0,387542 | 8,470753 | 12,08164 | 6,32E-31 | 8,57E-29 | 59,39449 |
| FNDC1 | 1,09584 | 7,481561 | 12,06727 | 7,33E-31 | 9,82E-29 | 59,24923 |
| DPYSL3 | 0,687898 | 8,008495 | 12,05279 | 8,5E-31 | 1,13E-28 | 59,10304 |
| FHL3 | 0,362295 | 6,149628 | 12,04226 | 9,46E-31 | 1,24E-28 | 58,99677 |
| PLOD1 | 0,433945 | 7,512555 | 12,04155 | 9,53E-31 | 1,24E-28 | 58,98962 |
| MAP4K4 | 0,376868 | 7,340825 | 12,02849 | 1,09E-30 | 1,4E-28 | 58,85788 |
| THBS2 | 0,980108 | 8,677321 | 12,02512 | 1,13E-30 | 1,43E-28 | 58,82388 |
| SH3PXD2B | 0,463228 | 7,059757 | 12,01985 | 1,19E-30 | 1,5E-28 | 58,77079 |
| HTRA1 | 0,673664 | 8,998871 | 11,99317 | 1,56E-30 | 1,95E-28 | 58,50217 |
| KIAA1958 | -0,32564 | 4,424095 | -11,9726 | 1,93E-30 | 2,38E-28 | 58,29554 |
| TGFBI | 0,71489 | 8,3599 | 11,96346 | 2,11E-30 | 2,58E-28 | 58,20347 |
| TIMP3 | 0,734391 | 8,944193 | 11,94689 | 2,5E-30 | 3,03E-28 | 58,03715 |
| NOX4 | 0,799875 | 5,647108 | 11,89514 | 4,23E-30 | 5,07E-28 | 57,51872 |
| PACS1 | 0,306081 | 7,234809 | 11,88735 | 4,58E-30 | 5,41E-28 | 57,4407 |
| CTSA | 0,335783 | 8,551095 | 11,88691 | 4,6E-30 | 5,41E-28 | 57,4364 |
| FMNL1 | 0,462211 | 5,772626 | 11,8463 | 6,94E-30 | 8,08E-28 | 57,03079 |
| ITGA5 | 0,602724 | 7,521628 | 11,83212 | 8E-30 | 9,24E-28 | 56,88939 |
| RAI14 | 0,485848 | 6,791047 | 11,83084 | 8,11E-30 | 9,27E-28 | 56,87657 |
| CHPF | 0,447576 | 7,107588 | 11,79592 | 1,15E-29 | 1,31E-27 | 56,52886 |
| DBN1 | 0,44605 | 7,423918 | 11,78005 | 1,35E-29 | 1,52E-27 | 56,37114 |
| CAPZB | 0,322572 | 8,338645 | 11,76759 | 1,53E-29 | 1,71E-27 | 56,24736 |
| CCM2 | 0,271192 | 6,555791 | 11,7483 | 1,86E-29 | 2,06E-27 | 56,05593 |
| CNPY4 | 0,366172 | 5,334492 | 11,74136 | 2E-29 | 2,18E-27 | 55,98712 |
| RHOG | 0,321308 | 7,265132 | 11,71531 | 2,59E-29 | 2,81E-27 | 55,72901 |
| TBC1D2 | 0,556571 | 6,350846 | 11,70543 | 2,86E-29 | 3,08E-27 | 55,63125 |
| MFAP2 | 0,649416 | 6,685415 | 11,6986 | 3,07E-29 | 3,27E-27 | 55,56369 |
| VAT1 | 0,340093 | 7,910264 | 11,69539 | 3,17E-29 | 3,35E-27 | 55,53193 |
| FBN1 | 0,817158 | 8,255082 | 11,67025 | 4,08E-29 | 4,27E-27 | 55,28356 |
| GGT5 | 0,514708 | 7,407798 | 11,66068 | 4,48E-29 | 4,66E-27 | 55,18914 |
| FRMD6 | 0,579976 | 6,24654 | 11,61834 | 6,84E-29 | 7,05E-27 | 54,77188 |
| PTK7 | 0,540097 | 7,002695 | 11,59878 | 8,32E-29 | 8,5E-27 | 54,57955 |
| PRSS23 | 0,563628 | 7,690401 | 11,5297 | 1,65E-28 | 1,68E-26 | 53,90188 |
| TNFAIP6 | 0,803861 | 5,884283 | 11,50646 | 2,08E-28 | 2,09E-26 | 53,67457 |
| FBXO32 | 0,672348 | 7,518801 | 11,49558 | 2,32E-28 | 2,31E-26 | 53,5682 |
| LIMK1 | 0,356401 | 6,905663 | 11,47876 | 2,74E-28 | 2,71E-26 | 53,40404 |
| BASP1 | 0,513478 | 7,511362 | 11,45023 | 3,63E-28 | 3,56E-26 | 53,12584 |
| CTSK | 0,782174 | 8,864678 | 11,44733 | 3,74E-28 | 3,64E-26 | 53,09762 |
| HEXA | 0,272548 | 6,59435 | 11,42892 | 4,48E-28 | 4,33E-26 | 52,91847 |
| GRN | 0,46749 | 9,707929 | 11,39733 | 6,12E-28 | 5,86E-26 | 52,61146 |
| TRAM2 | 0,370789 | 6,652802 | 11,36915 | 8,07E-28 | 7,68E-26 | 52,33803 |
| ADAM12 | 0,80846 | 5,792392 | 11,36774 | 8,18E-28 | 7,73E-26 | 52,32441 |
| RBMS1 | 0,359715 | 7,05923 | 11,34812 | 9,92E-28 | 9,3E-26 | 52,13437 |
| PCOLCE | 0,590373 | 7,707831 | 11,33272 | 1,15E-27 | 1,07E-25 | 51,98544 |
| ID3 | 0,45994 | 6,783216 | 11,31658 | 1,35E-27 | 1,25E-25 | 51,82942 |
| ANTXR1 | 0,665735 | 7,531433 | 11,30643 | 1,49E-27 | 1,37E-25 | 51,73139 |
| FMNL3 | 0,355378 | 6,032568 | 11,30429 | 1,52E-27 | 1,39E-25 | 51,71072 |
| ACTN1 | 0,444831 | 8,989481 | 11,29918 | 1,6E-27 | 1,45E-25 | 51,66139 |
| WIPF1 | 0,468366 | 6,937047 | 11,25199 | 2,54E-27 | 2,28E-25 | 51,2068 |
| EML3 | 0,289415 | 6,338604 | 11,22755 | 3,22E-27 | 2,87E-25 | 50,97189 |
| LNX1 | -0,38507 | 5,081994 | -11,2261 | 3,27E-27 | 2,89E-25 | 50,95819 |
| NRG4 | -0,76113 | 4,154219 | -11,2188 | 3,51E-27 | 3,08E-25 | 50,88819 |
| MMP14 | 0,609854 | 8,444366 | 11,21748 | 3,56E-27 | 3,1E-25 | 50,87515 |
| ITPRIP | 0,447255 | 6,762842 | 11,20922 | 3,85E-27 | 3,34E-25 | 50,79591 |
| CPZ | 0,642369 | 5,155041 | 11,20066 | 4,19E-27 | 3,6E-25 | 50,71375 |
| NAGK | 0,295205 | 7,497258 | 11,18951 | 4,67E-27 | 3,99E-25 | 50,60688 |
| ARSI | 0,559581 | 4,590242 | 11,18733 | 4,77E-27 | 4,04E-25 | 50,58599 |
| JOSD1 | 0,261818 | 7,272593 | 11,18533 | 4,86E-27 | 4,1E-25 | 50,56682 |
| HOOK1 | -0,52426 | 5,208016 | -11,1832 | 4,96E-27 | 4,15E-25 | 50,54633 |
| AFAP1 | 0,427947 | 6,206989 | 11,18138 | 5,05E-27 | 4,2E-25 | 50,52906 |
| CEBPB | 0,436422 | 8,445899 | 11,16967 | 5,66E-27 | 4,67E-25 | 50,4169 |
| COL12A1 | 0,902136 | 8,403299 | 11,16578 | 5,88E-27 | 4,82E-25 | 50,37975 |
| TLE3 | 0,264648 | 6,555769 | 11,14551 | 7,15E-27 | 5,83E-25 | 50,18593 |
| SPHK1 | 0,572865 | 6,570297 | 11,11639 | 9,48E-27 | 7,68E-25 | 49,90799 |
| MPV17 | 0,255542 | 7,059013 | 11,11371 | 9,73E-27 | 7,83E-25 | 49,88242 |
| THBS1 | 0,77155 | 8,958773 | 11,09579 | 1,16E-26 | 9,25E-25 | 49,71166 |
| RCN1 | 0,392621 | 7,39943 | 11,09439 | 1,17E-26 | 9,31E-25 | 49,69832 |
| PANX1 | 0,391072 | 6,226649 | 11,08676 | 1,26E-26 | 9,96E-25 | 49,62565 |
| PLEKHM2 | 0,270551 | 6,575476 | 11,06926 | 1,49E-26 | 1,17E-24 | 49,4592 |
| PMP22 | 0,555892 | 8,359925 | 11,06634 | 1,54E-26 | 1,2E-24 | 49,43144 |
| AP2S1 | 0,313505 | 7,206038 | 11,05331 | 1,74E-26 | 1,35E-24 | 49,30767 |
| ADAMTS2 | 0,490132 | 6,864555 | 11,03616 | 2,06E-26 | 1,58E-24 | 49,14482 |
| MYL6 | 0,352082 | 10,1527 | 11,03377 | 2,1E-26 | 1,61E-24 | 49,12221 |
| ACTG1 | 0,335175 | 11,22101 | 10,99038 | 3,19E-26 | 2,43E-24 | 48,71126 |
| MGAT1 | 0,260742 | 7,765932 | 10,98807 | 3,26E-26 | 2,45E-24 | 48,68939 |
| NID2 | 0,6794 | 6,43482 | 10,98796 | 3,27E-26 | 2,45E-24 | 48,68833 |
| GAS2 | -0,58761 | 4,031921 | -10,9634 | 4,14E-26 | 3,09E-24 | 48,45602 |
| TPD52L2 | 0,318377 | 6,845644 | 10,96093 | 4,23E-26 | 3,14E-24 | 48,43297 |
| ADAMTS12 | 0,592713 | 5,963723 | 10,93197 | 5,58E-26 | 4,12E-24 | 48,15986 |
| PEA15 | 0,336354 | 8,310451 | 10,92825 | 5,79E-26 | 4,24E-24 | 48,12487 |
| NOTCH3 | 0,368957 | 6,966056 | 10,92522 | 5,96E-26 | 4,34E-24 | 48,09634 |
| APBB2 | 0,370122 | 6,814041 | 10,91461 | 6,59E-26 | 4,78E-24 | 47,99648 |
| EDNRA | 0,637297 | 6,80564 | 10,90728 | 7,07E-26 | 5,09E-24 | 47,92745 |
| ETS1 | 0,402701 | 7,358439 | 10,90047 | 7,54E-26 | 5,4E-24 | 47,86346 |
| AP2A1 | 0,254864 | 6,575889 | 10,88914 | 8,4E-26 | 5,99E-24 | 47,75694 |
| PODNL1 | 0,515774 | 5,253792 | 10,88571 | 8,68E-26 | 6,15E-24 | 47,72476 |
| HOMER3 | 0,385435 | 6,809265 | 10,88519 | 8,73E-26 | 6,15E-24 | 47,71984 |
| SPTAN1 | 0,299599 | 8,1313 | 10,87021 | 1,01E-25 | 7,05E-24 | 47,57927 |
| GRINA | 0,28852 | 7,307472 | 10,86583 | 1,05E-25 | 7,31E-24 | 47,53818 |
| SFRP2 | 0,977218 | 9,412937 | 10,82603 | 1,53E-25 | 1,06E-23 | 47,16536 |
| RIN3 | 0,289586 | 5,836877 | 10,80013 | 1,96E-25 | 1,35E-23 | 46,9233 |
| CRIP2 | 0,481898 | 7,610968 | 10,79814 | 1,99E-25 | 1,37E-23 | 46,90475 |
| CTSD | 0,44579 | 9,213221 | 10,79285 | 2,1E-25 | 1,43E-23 | 46,85539 |
| SMARCB1 | 0,25162 | 6,376962 | 10,77279 | 2,54E-25 | 1,72E-23 | 46,66833 |
| FTL | 0,391751 | 10,6906 | 10,76999 | 2,6E-25 | 1,75E-23 | 46,64216 |
| ARPC2 | 0,303914 | 9,710751 | 10,75305 | 3,06E-25 | 2,05E-23 | 46,48445 |
| ARHGDIA | 0,266596 | 7,658104 | 10,74208 | 3,39E-25 | 2,26E-23 | 46,3824 |
| NT5DC2 | 0,382998 | 6,253941 | 10,72435 | 4,01E-25 | 2,66E-23 | 46,2176 |
| VEGFC | 0,508354 | 5,82247 | 10,70964 | 4,6E-25 | 3,04E-23 | 46,08103 |
| MXRA5 | 0,675142 | 8,929323 | 10,70753 | 4,69E-25 | 3,08E-23 | 46,06143 |
| CHDH | -0,47526 | 5,962087 | -10,6774 | 6,23E-25 | 4,07E-23 | 45,78228 |
| ABCC6 | -0,42474 | 4,831325 | -10,67 | 6,68E-25 | 4,34E-23 | 45,71414 |
| PLD3 | 0,289207 | 7,549481 | 10,64638 | 8,34E-25 | 5,39E-23 | 45,49527 |
| GPX4 | 0,32361 | 8,534505 | 10,64418 | 8,51E-25 | 5,47E-23 | 45,47495 |
| PLAUR | 0,587951 | 7,697848 | 10,64034 | 8,82E-25 | 5,64E-23 | 45,43953 |
| SH3PXD2A | 0,423417 | 7,24586 | 10,61746 | 1,09E-24 | 6,95E-23 | 45,2284 |
| LOXL3 | 0,327509 | 5,171592 | 10,61493 | 1,12E-24 | 7,05E-23 | 45,20508 |
| TLN1 | 0,350684 | 7,981048 | 10,6149 | 1,12E-24 | 7,05E-23 | 45,20477 |
| CHSY1 | 0,406661 | 7,422982 | 10,60874 | 1,19E-24 | 7,43E-23 | 45,14798 |
| PKN1 | 0,291172 | 7,005294 | 10,60616 | 1,21E-24 | 7,57E-23 | 45,12423 |
